# Loss of INPP5E affects photoreceptor outer segment membrane biogenesis in iPSC-derived human retinal organoids

**DOI:** 10.1242/jcs.264431

**Published:** 2026-07-21

**Authors:** Kae R. Whiting, Mariam G. Aslanyan, Lynn van Summeren, Tina Beyer, Katrin Dahlke, Thomas Theil, Karsten Boldt, Ronald Roepman

**Affiliations:** ^1^ Department of Human Genetics, Research Institute for Medical Innovation, Radboud University Medical Center, Nijmegen 6525 GA, The Netherlands; ^2^ Eberhard Karls University of Tübingen, Institute for Ophthalmic Research, Tübingen 72074, Germany; ^3^ Eberhard Karls University of Tübingen, Core Facility for Medical Proteomics, Tübingen 72074, Germany; ^4^ Institute for Neuroscience and Cardiovascular Sciences, University of Edinburgh, Hugh Robson Building, Edinburgh EH8 9XD, UK; ^5^ Simons Initiative for the Developing Brain, University of Edinburgh, Hugh Robson Building, Edinburgh EH8 9XD, UK

**Keywords:** Retinal organoids, INPP5E, Membrane biogenesis, Retinal development, Retinal ciliopathies, Cilia

## Abstract

Mutations in the ciliary gene *INPP5E*, encoding inositol polyphosphate-5-phosphatase E (INPP5E), can cause retinal degeneration as part of the ciliopathy Joubert syndrome or non-syndromic retinitis pigmentosa (RP). INPP5E regulates the membrane makeup of the primary cilium; however, its function in the specialized sensory photoreceptor cells of the human retina remain unclear. Here, we utilize control and CRISPR/Cas9-generated *INPP5E* knockout (*INPP5E^D477N/D477N^*) human induced pluripotent stem cells (iPSCs) to generate retinal organoids (ROs). Through proteomic and immunofluorescence analysis, we show that INPP5E plays an important role in early retinal development and photoreceptor progenitor cell differentiation. In mature ROs, INPP5E localizes to the connecting cilium of photoreceptors, and the loss of INPP5E leads to altered localization of ARL13B and rhodopsin in mature photoreceptors. Furthermore, photoreceptor outer segment structure is affected, leading to elongated outer segment membranes in both cone and rod photoreceptors, suggesting an important role for INPP5E in photoreceptor outer segment membrane biogenesis. Together, these data underline the importance of INPP5E in retina development and photoreceptor structure and highlight the usability of ROs to study protein function in a human context.

## Teaser

**Summary:** Human retinal organoids with disruption of INPP5E-dependent phosphoinositide homeostasis show remodeling of axonemal architecture and outer segment membrane biogenesis of photoreceptor sensory cilia.

## INTRODUCTION

Photoreceptors in the retina carry highly specialized primary cilia that play a vital role in light detection and transduction. Primary cilia are small, hair-like and non-motile sensory organelles that are important during organismal development and tissue homeostasis (Mill et al., 2023); however, the photoreceptor sensory cilium has a unique structure – including an extended transition zone called the connecting cilium (CC) and an outer segment (OS) filled with opsin-loaded outer segment discs – that allows the photoreceptor sensory cilium to function as a signal transducer during the phototransduction cascade (Wensel et al., 2021). Pathogenic variants in genes encoding proteins that are important for photoreceptor function can cause inherited retinal degeneration (IRD), such as retinitis pigmentosa (RP), which affects around one in 4000 individuals worldwide (Cross et al., 2022; Hamel, 2006). RP is a progressive disorder characterized by the gradual loss of photoreceptors in the retina, initially presenting as night blindness followed by the progressive loss of vision (Hamel, 2006; Verbakel et al., 2018). To date, upwards of 80 genes have been identified as RP-causative genes (see https://retnet.org/), with manifestations of the disorder occurring as both non-syndromic, where RP is the only phenotype, and syndromic disorders that affect multiple organs alongside the retina. Ciliopathies are a class of developmental disorders caused by defects in the function of the cilium, with RP being a prominent phenotype in many ciliopathies (Chen et al., 2019).

One gene that has been found to cause both syndromic and non-syndromic RP when mutated is *INPP5E* (Sangermano et al., 2021; Whiting et al., 2024), which encodes the protein inositol polyphosphate-5-phosphatase E (INPP5E), a phosphatase important for regulating the phospholipid membrane of the primary cilium by cleaving the 5′ phosphate group from the phosphoinositide phosphatidylinositol 4,5-bisphosphate [PI(4,5)P_2_] to form phosphatidylinositol 4-phosphate [PI(4)P] (Chávez et al., 2015; Garcia-Gonzalo et al., 2015; Phua et al., 2017). In doing so, INPP5E plays an important role in organismal development and tissue homeostasis by regulating signaling pathways (Bangs and Anderson, 2017; Gigante et al., 2020; Zhang et al., 2022), cell cycle entry (Phua et al., 2017; Plotnikova et al., 2009; Sierra Potchanant et al., 2017) and neural patterning during early brain development (Schembs et al., 2022). Pathogenic variants in *INPP5E* can lead to several disorders including MORM syndrome (OMIM #610156), Joubert syndrome (JS, OMIM #213300), Leber congenital amaurosis (LCA, MIM:PS204000), and non-syndromic retinitis pigmentosa (RP, OMIM #268000) (Bielas et al., 2009; Jacoby et al., 2009; Sangermano et al., 2021; Wang et al., 2013, 2015).

To date, the role of INPP5E in photoreceptors has only been studied using mouse models. INPP5E localizes to the inner segment and connecting cilium of photoreceptors where it plays a role in protein trafficking and OS disc stability, and retinal specific deletion of *Inpp5e* causes rapid degeneration of photoreceptor OSs (Gupta et al., 2025; Sharif et al., 2021). However, mouse models do not always recapitulate the human phenotype when modeling retinal dysfunction (Moshiri, 2021; Slijkerman et al., 2015). To better understand the role of INPP5E in the human retina, we used human induced pluripotent stem cells (iPSCs) containing a CRISPR/Cas9-generated point mutation (D477N) that renders the catalytic domain of INPP5E non-functional, leading to a functional knockout of INPP5E [*INPP5E^D477N/D477N^*; generated and characterized previously in Schembs et al. (2022)] to generate retinal organoids (ROs). We subsequently used these ROs to model the function of INPP5E in human retina development and photoreceptor maintenance.

The *INPP5E^D477N/D477N^* ROs show a delay in photoreceptor precursor generation at early organoid timepoints, but this delay does not hinder the generation of more-mature photoreceptors. We confirm the localization of INPP5E in the connecting cilia of wild-type photoreceptors but could not detect INPP5E in photoreceptors of *INPP5E^D477N/D477N^* ROs. In mature ROs, there is an increase in the number of rod and cone photoreceptors, and the structural morphology of both rod and cone photoreceptor OSs is altered by the loss of INPP5E, leading to longer OSs compared to control organoids. Finally, we show that ARL13B and rhodopsin are mislocalized in *INPP5E^D477N/D477N^* ROs. Together, this indicates a complex and crucial function for INPP5E during retina development and photoreceptor maintenance.

## RESULTS

### *INPP5E^D477N/D477N^* iPSCs differentiate into mature ROs

**Fig. 1.**
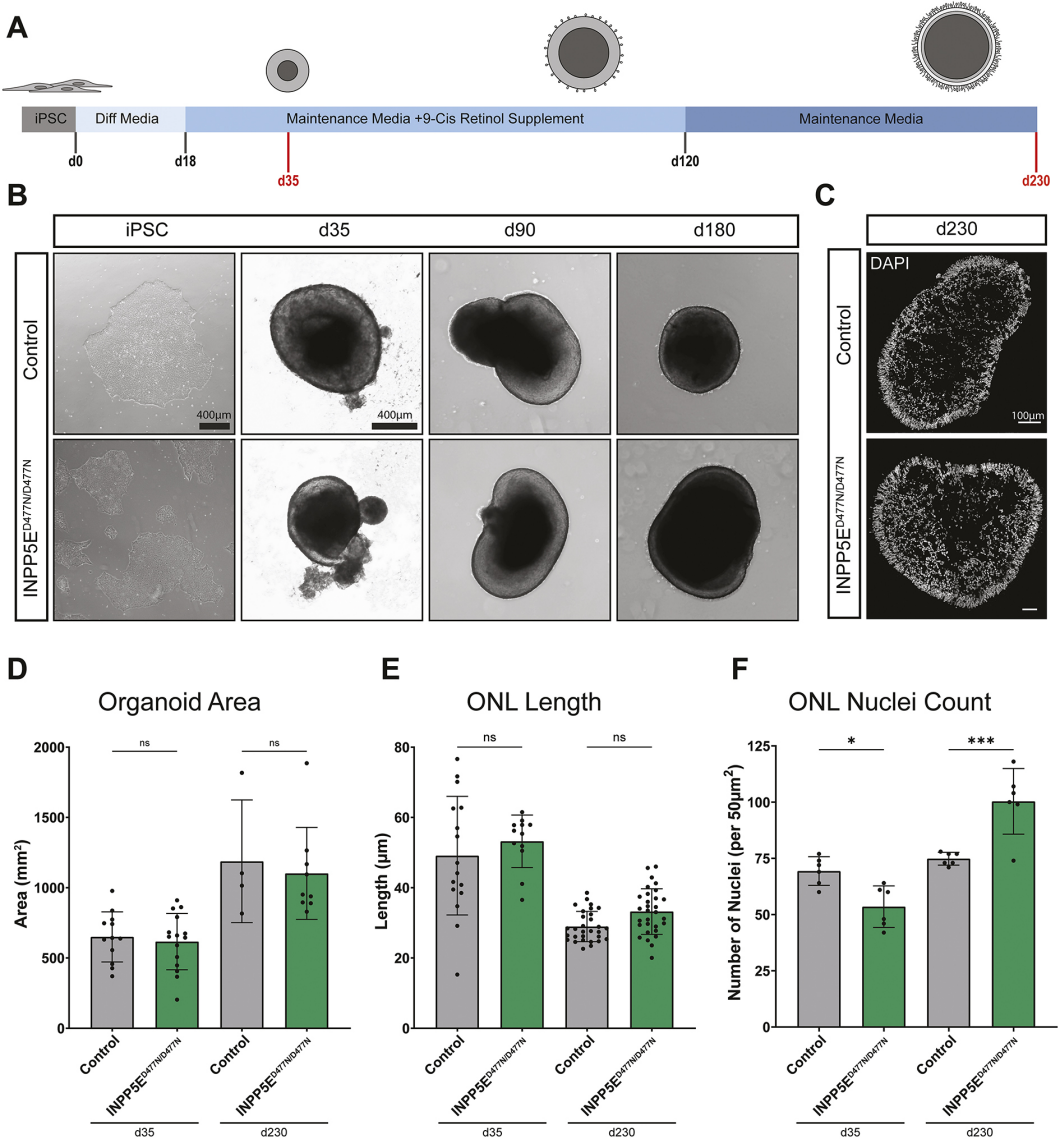
**Differentiation of iPSCs to ROs.** (A) Protocol overview of the method to differentiate iPSCs into ROs. The composition of differentiation (Diff) and maintenance media can be found in methods. Organoids were collected at d35 and d230 for immunofluorescence (IF) and proteomic analysis. (B) Brightfield images of organoids through development. Control and *INPP5E^D477N/D477N^* organoids show no major morphological changes. Scale bars: 400 μm. (C) IF of mature organoids at d230 stained for DAPI. Scale bars: 100 μm. (D–F) Quantification of basic RO morphology at early (d35) and late (d230) stages of development as (D) RO area, (E) outer nuclear layer (ONL) length, and (F) quantity of DAPI in the ONL as the mean±s.d. (n=6 organoids). Significance was determined by one-way ANOVA with post-hoc Tukey's test (*P=0.0371; ***P=0.0007; ns, not significant).

To study the role of INPP5E in human retina development and photoreceptor function, we differentiated both control and *INPP5E^D477N/D477N^* iPSCs into ROs over the course of 230 days (Fig. 1A). Organoid morphology was tracked throughout development via brightfield microscopy, which showed proper RO development in both control and *INPP5E^D477N/D477N^* ROs (Fig. 1B). Organoid area was measured at day (d)35 and d230 based on brightfield images, where there was no difference between control and mutant organoids (Fig. 1D). Through immunofluorescence staining of cryopreserved and sectioned organoids, the retinal progenitor and retinal cell containing outer nuclear layer (ONL) could be visualized (Fig. 1C) and quantified (Fig. 1E; Fig. S5). No difference in ONL length between developing (d35) and mature (d230) organoids was seen. Interestingly, though, there was a difference between the number of nuclei in the ONL of control and *INPP5E^D477N/D477N^* organoids (Fig. 1F). At d35, there was a slight decrease in the number of nuclei per 50 μm^2^ in *INPP5E^D477N/D477N^* organoids (53.5±9.2 nuclei/50 μm^2^; mean±s.d.) compared to the control (69.3±6.4 nuclei/50 μm^2^). However, at d230, the number of nuclei per 50 μm^2^ significantly increased in *INPP5E^D477N/D477N^* ROs compared to that in control (control: 74.8±2.9 nuclei/50 μm^2^; *INPP5E^D477N/D477N^*: 100.3±14.6 nuclei/50 μm^2^).

**Fig. 2.**
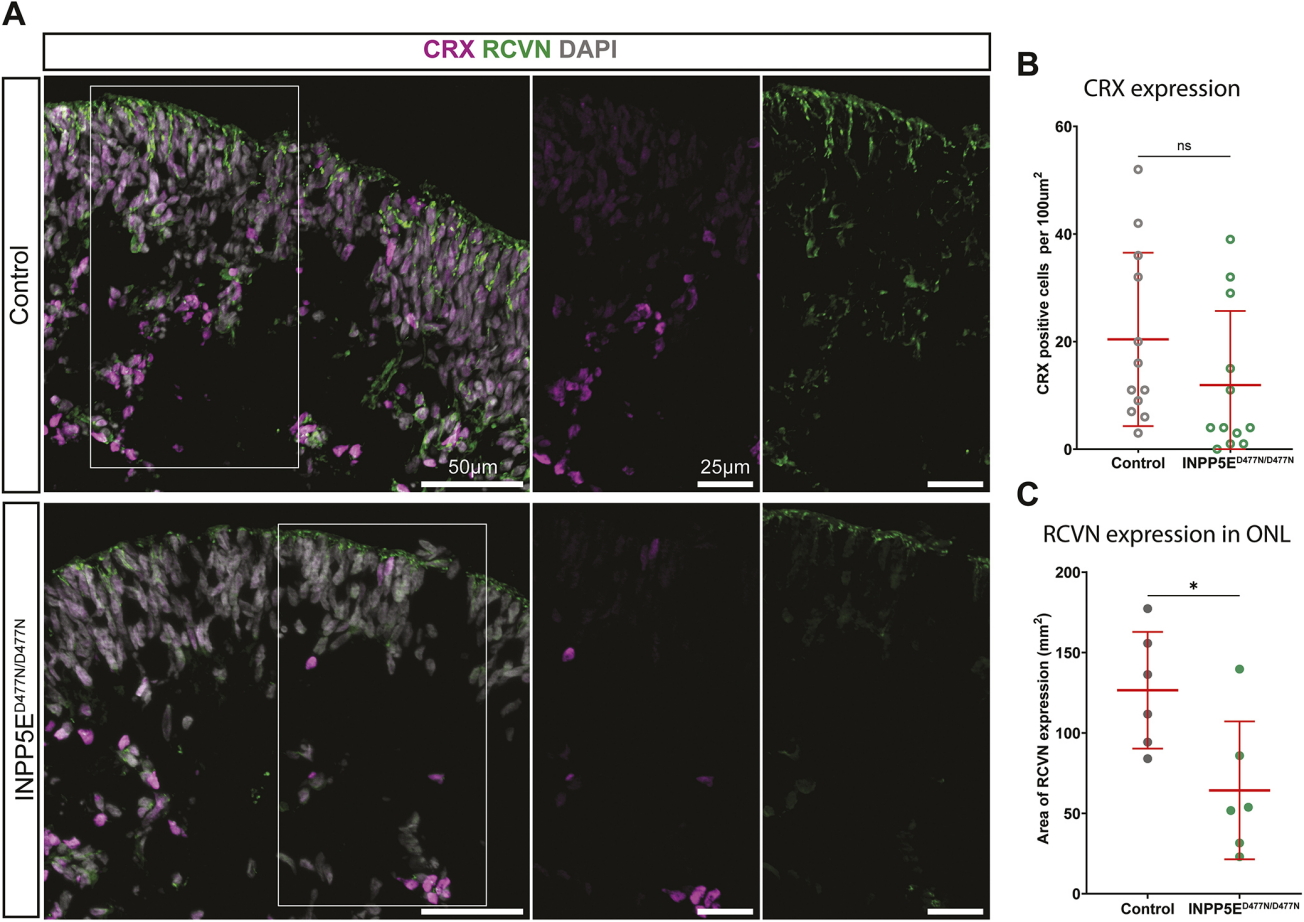
***INPP5E^D477N/D477N^* organoids have delayed expression of photoreceptor precursor markers.** (A) Representative immunofluorescence of retinal precursor markers CRX (magenta) and RCVN (green) at d35 of differentiation. Scale bars: 50 μm and 25 μm. (B,C) Quantification of photoreceptor precursor markers as the percentage of CRX-positive nuclei (B) and area of RCVN expression in the ONL (C), presented as the mean±s.d. (*n*=6 organoids). Significance was determined by a two-tailed unpaired *t*-test (*P<0.05; ns, not significant).

Furthermore, we used immunofluorescence analysis of the photoreceptor precursor markers recoverin (RCVN) and CRX to confirm the differentiation towards ROs and to determine whether there are any phenotypic differences between control and knockout organoids in early development (Fig. 2A). At early stages of control RO development (d35), the photoreceptor precursor markers RCVN and CRX are initially expressed. *INPP5E^D477N/D477N^* organoids showed a delay in photoreceptor precursor expression. Compared to control, *INPP5E^D477N/D477N^* ROs had a slight, but not significant, decrease in the percentage of CRX-positive nuclei (control, 20.42%; *INPP5E^D477N/D477N^*, 11.92%) and a significant change in RCVN expression in the ONL [control, 126.5±36 arbitrary units (A.U.); *INPP5E^D477N/D477N^*, 64.28±43 A.U.; mean±s.d.] (Fig. 2B,C). Interestingly, by d230 of differentiation, no difference between CRX or RCVN expression is present (Fig. S1).

### Proteomics of ROs confirms retinal fate

**Fig. 3.**
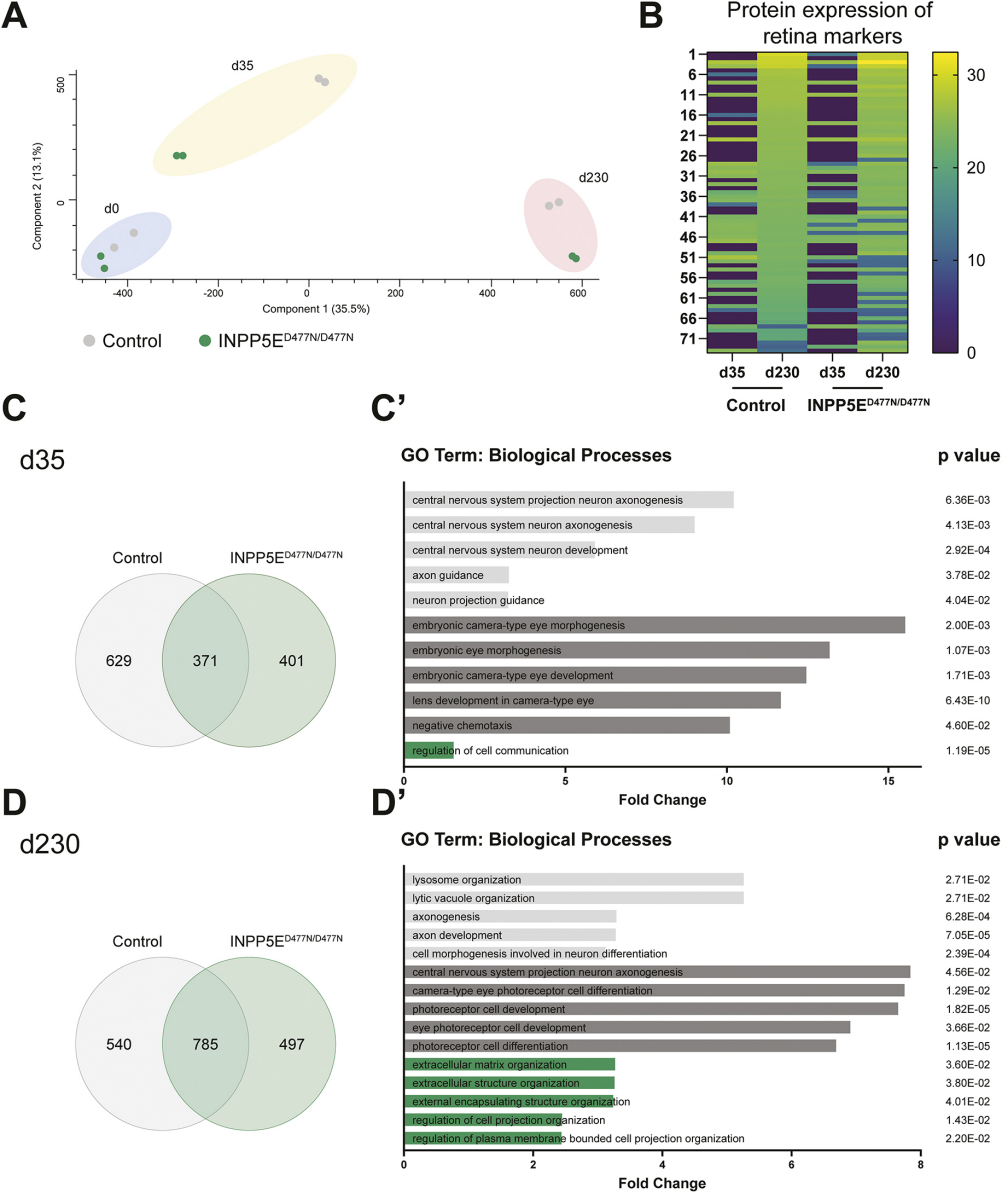
**Bulk proteomics of organoids confirms retinal fate.** (A) PCA plot of d0, d35 and d230 organoids showing a trajectory of development as organoids differentiate further from iPSCs. (B) Protein expression of retina markers increases from d35 to d230 as organoids develop in both control and *INPP5E^D477N/D477N^* ROs. Comparison of enriched protein datasets at d35 (C) and d230 (D) of organoid development show high protein overlap at d230. (C′,D′) Top GO terms for biological processes of enriched proteins at d35 and d230. GO terms for proteins enriched in control organoids are light gray, in only *INPP5E^D477N/D477N^* organoids in green and in both datasets in dark gray.

To confirm retinal differentiation of iPSCs to ROs, bulk proteomics was performed on undifferentiated iPSCs (d0), early stage ROs (d35) and mature ROs (d230) (Table S2). Principal component analysis (PCA) revealed distinct clusters based on differentiation stage, demonstrating that mature ROs cluster further apart from iPSCs (Fig. 3A). Additionally, the expression of retinal specific markers – such as ARR3, RHO and PRPH2 – markedly increased from d35 to d230 (Fig. 3B).

To better understand the mechanism of action for INPP5E within the ROs, Gene Ontology (GO) enrichment analysis for biological processes (BP) was performed. Proteins were classified into three groups: proteins enriched in only control ROs (light gray), proteins enriched in only *INPP5E^D477N/D477N^* ROs (green) and proteins enriched in both datasets (dark gray) (Fig. 3C,D). During early RO differentiation (d35) several BP terms for embryonic eye development were enriched in both control and *INPP5E^D477N/D477N^* ROs. BP terms for axon guidance and axonogenesis were highly enriched in control organoids, while in *INPP5E^D477N/D477N^* ROs regulation of cell communication was the only significant term (Fig. 3C′). In mature ROs (d230), BP terms for photoreceptor cell development were highly enriched in both datasets. However, there was a difference in GO terms identified for control and *INPP5E^D477N/D477N^* ROs: control ROs were enriched for axonogenesis and lysosome organization while *INPP5E^D477N/D477N^* ROs were enriched for cell projection organization and extracellular matrix organization (Fig. 3D′).

### Cilia morphology and INPP5E expression is affected in *INPP5E^D477N/D477N^* ROs

**Fig. 4.**
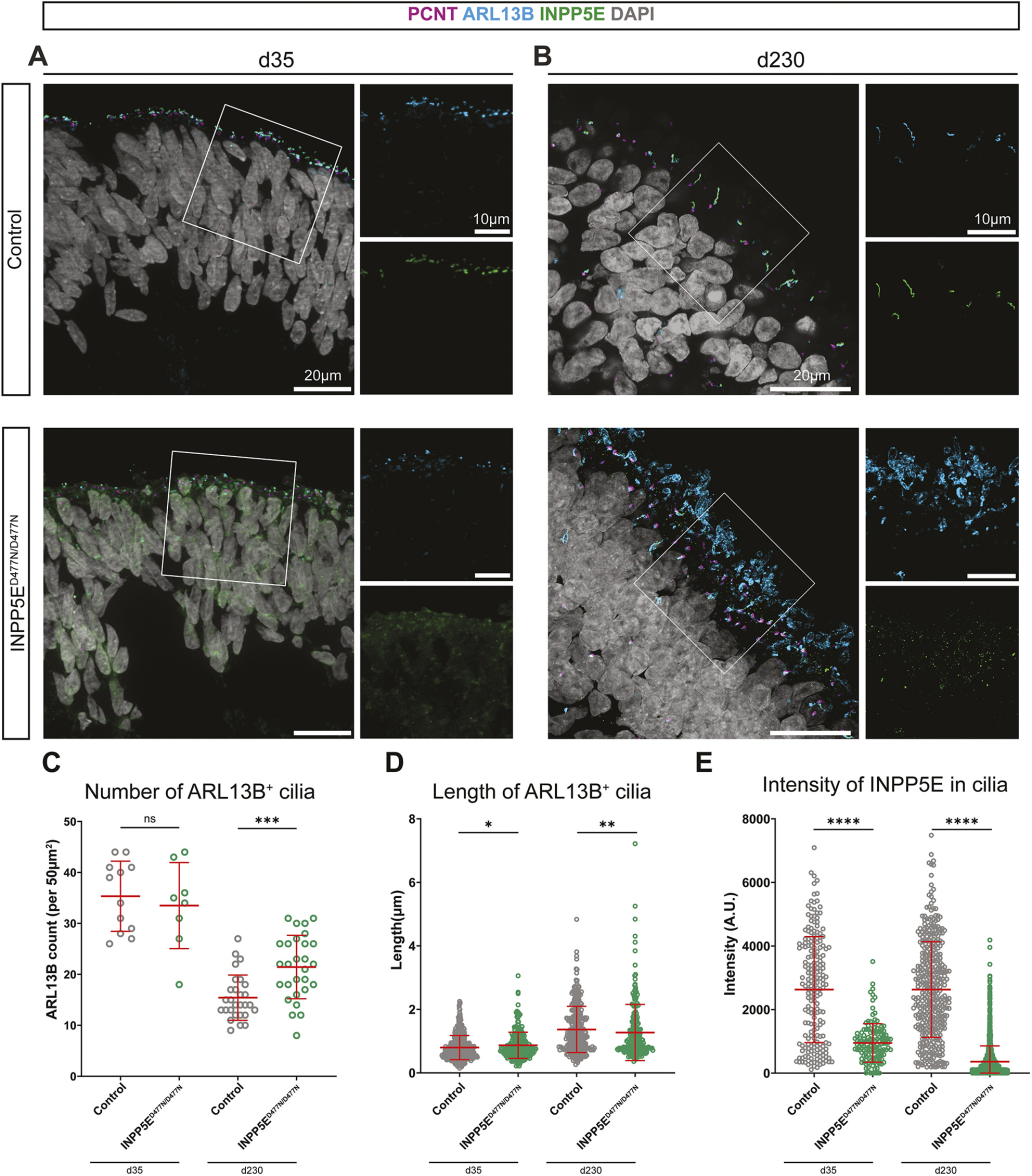
**Structural changes of primary and connecting cilia in *INPP5E^D477N/D477N^* organoids.** (A,B) Representative immunofluorescence images of d35 (A) and d230 (B) ROs stained for the basal body marker PCNT (magenta), the ciliary membrane marker ARL13B (blue), DAPI (gray) and INPP5E (green). (C–E) Quantification of ciliary morphology and INPP5E localization as the number of ARL13B^+^ cilia per 50 μm^2^ (C), length of cilia (μm) (D) and intensity of INPP5E expression in the cilia (A.U.) (E), presented as the mean±s.d. (*n*=6 organoids). Significance was determined by one-way ANOVA with post-hoc Tukey's test (*P=0.0139; **P=0.0083; ***P=0.0001; ****P<0.0001; ns, not significant).

In most cell models, ARL13B regulates INPP5E localization at the ciliary membrane of the primary cilium, where the two colocalize (Qiu et al., 2021). In the retina, INPP5E localizes to the inner segment and connecting cilium of photoreceptors in mouse, non-human primate and the mature human macula (Sharif et al., 2021). Moreover, previous studies have shown that defects in INPP5E, due to either specific disease-causing variants or CRISPR/Cas9-generated knockouts, lead to both the mislocalization of INPP5E to the cilium and shorter cilia (Schembs et al., 2022; Whiting et al., 2024). As INPP5E colocalizes with ARL13B in the primary cilium, here we analyzed the fluorescence intensity of INPP5E in ARL13B^+^ cilia to compare the localization of INPP5E in both developing and mature ROs. In control organoids, INPP5E colocalized with ARL13B along the primary cilium of both d35 ROs (2627±1669 A.U.) and mature d230 ROs (2629±1505 A.U.; mean±s.d.). In *INPP5E^D477N/D477N^* organoids, INPP5E expression was significantly decreased at both d35 and d230 of RO development (d35, 948.1±605 A.U.; d230, 358.7±496.5 A.U.) (Fig. 4E).

Next, to determine whether the loss of INPP5E affects ciliary morphology during RO differentiation, we utilized immunofluorescence to visualize the ciliary markers pericentrin (PCNT) and ARL13B in d35 and d230 ROs (Fig. 4A,B). In control ROs both the number and structure of cilia in control ROs changed over the course of differentiation. At d35, there were more cilia than at d230 (d35, 35±6.8 ARL13B^+^ cilia per 50 μm^2^; d230, 15±4.4 ARL13B^+^ cilia per 50 μm^2^; mean±s.d.) (Fig. 4C) whereas the length of cilia increased as the organoids developed and matured (d35: 0.797±0.38 μm; d230: 1.367±0.73 μm) (Fig. 4D).

*INPP5E^D477N/D477N^* organoids show slight defects in ciliary morphology during early development, with no significant differences in the number of cilia at d35 (33±8.4 ARL13B^+^ cilia per 50 μm^2^) (Fig. 4C) and a minor increase in cilia length compared to control (0.87±0.41 μm) (Fig. 4D). However, at d230 the number of ARL13B^+^ cilia significantly increased compared to control (21±6.2 ARL13B^+^ cilia per 50 μm^2^) whereas the length of the cilia decreased (1.27±0.88 μm) (Fig. 4C,D; Table 1).

**Table 1.** Results of ciliary phenotyping in d35 and d230 ROs

		Cilia count (per 50 μm^2^)	Cilia length (μm)	INPP5E localization (MFI, A.U.)
**d35**				
	Control	35±6.8	0.797±0.38	2627±1669
	*INPP5E^D477N/D477N^*	33±8.4	0.87±0.41	948.1±605
**d230**				
	Control	15±4.4	1.367±0.73	2629±1505
	*INPP5E^D477N/D477N^*	21±6.2	1.27±0.88	358.7±496.5

Data are as the mean±s.d. (*n*=6 organoids).

To determine whether other ciliary sub-compartments were affected, we used immunofluorescence of the basal body marker PCNT and the ciliary transition zone marker NPHP1. Expression of PCNT and NPHP1 was present at the basal region of the CC and no phenotypic alterations were apparent between control and *INPP5E^D477N/D477N^* ROs (Fig. S2).

### Photoreceptors of *INPP5E^D477N/D477N^* ROs have significant morphological defects

**Fig. 5.**
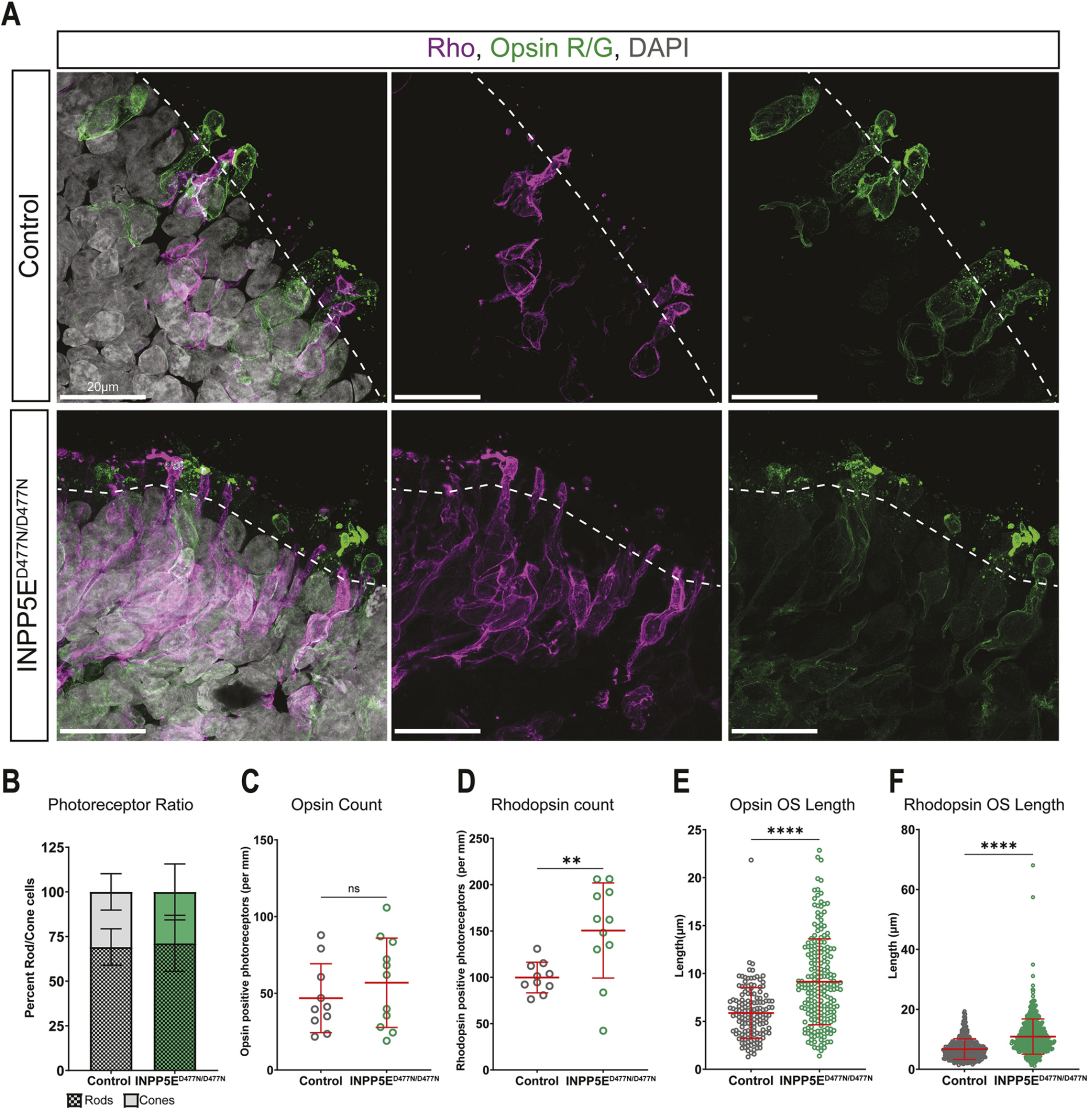
**Photoreceptor morphology is altered in *INPP5E^D477N/D477N^* organoids.** (A) Representative images of rod and cone photoreceptors in d230 organoids, stained for RHO (magenta), opsin R/G (green) and DAPI (gray). Dashed lines represent outer limiting membrane of organoids. Scale bars: 20 μm. (B) Quantification of rod:cone ratio in control and *INPP5E^D477N/D477N^* organoids as the percentage of RHO-positive and opsin-positive photoreceptors. (C,D) Quantification of photoreceptor development as the number of opsin positive (C) or rhodopsin positive (D) photoreceptors per mm. (E,F) Comparison of photoreceptor morphology as the length of the photoreceptor OS for opsin positive cone photoreceptors (E) and rhodopsin positive rod photoreceptors (F). All data presented as the mean±s.d. (*n*=6 organoids). Significance was determined by a two-tailed unpaired *t*-test (**P=0.0013; ****P<0.0001; ns, not significant).

To better understand the role of INPP5E in the development and maintenance of mature photoreceptors, we utilized immunofluorescence to visualize rod and cone photoreceptors in mature ROs, using rhodopsin (RHO) and opsin red/green (R/G), respectively (Fig. 5A). In control ROs, there were 100 RHO^+^ photoreceptors and 47 Opsin^+^ photoreceptors per mm, giving a 69:31% rod:cone photoreceptor ratio. In *INPP5E^D477N/D477N^* ROs, the ratio of rod-to-cone photoreceptors was not altered (71:29% rod:cone); however, there was an increase in the total number of both the rod and cone photoreceptors compared to control ROs (150 Rho^+^ photoreceptors per mm; 57 Opsin^+^ photoreceptors per mm) (Fig. 5B–D). Moreover, a significant increase in the length of the OSs in both cone (control: 5.87±2.6 μm; *INPP5E^D477N/D477N^*: 7.13±4.5 μm; mean±s.d.) and rod (control, 6.71±3.5 μm; *INPP5E^D477N/D477N^*, 10.90±5.9 μm) photoreceptors was observed in the *INPP5E^D477N/D477N^* ROs (Fig. 5E,F).

### Altered actin and tubulin regulation in *INPP5E^D477N/D477N^* organoids

**Fig. 6.**
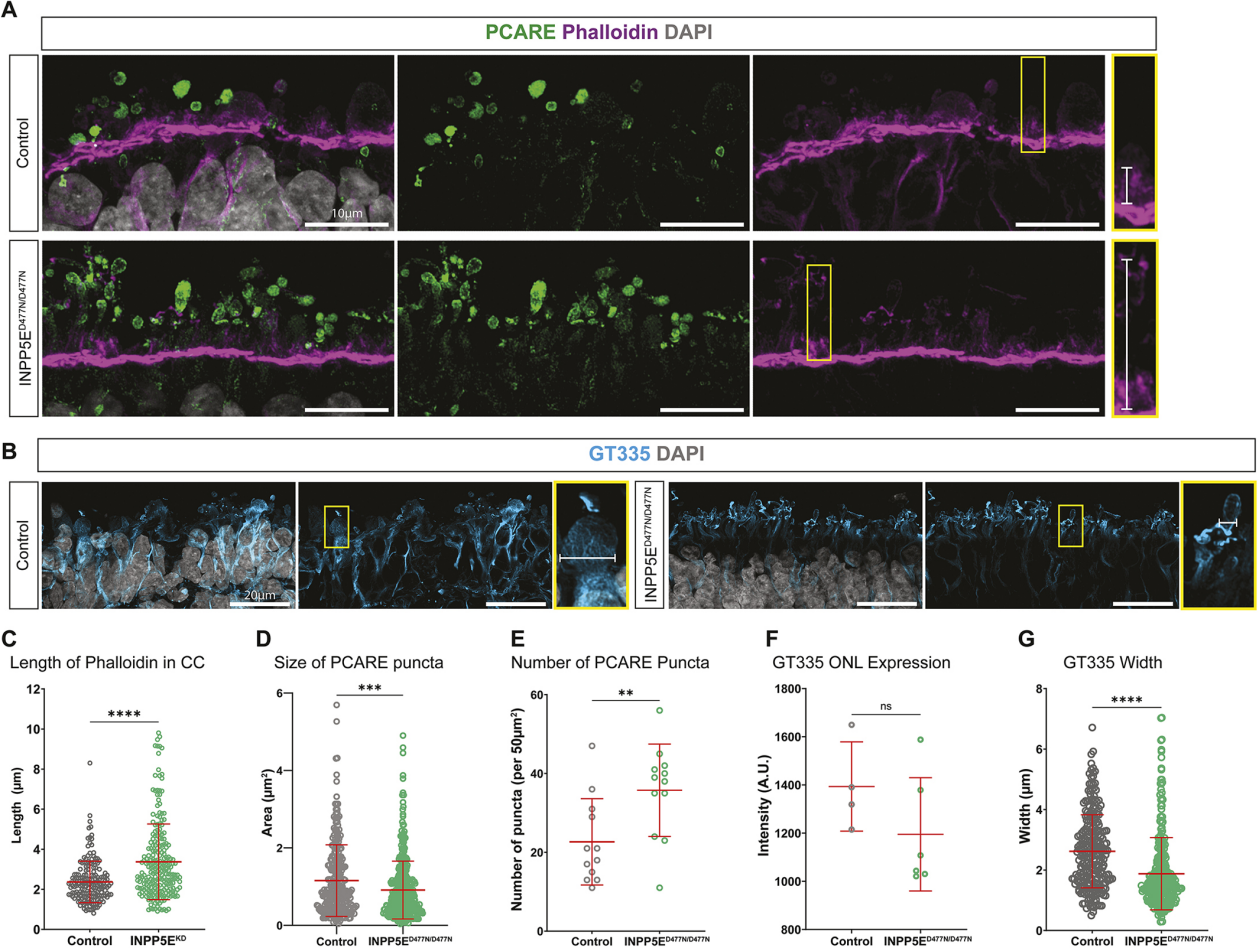
**Actin and tubulin dysregulation in d230 *INPP5E^D477N/D477N^* organoids.** (A) Representative immunofluorescence of F-actin (phalloidin, magenta), the actin regulator PCARE (green) and DAPI (gray) in d230 control and *INPP5E^D477N/D477N^* ROs. Yellow cutouts show examples of phalloidin extension into the photoreceptor connecting cilium. Scale bars: 10 μm. (B) Representative images of polyglutamylated tubulin (GT335, blue) and DAPI (gray) expression in d230 ROs. Cutout shows example of GT335 tubulin bundle measurement. Scale bars: 20 μm. Quantification of actin and actin regulators as the length of phalloidin extending into the CC (C), the size of PCARE puncta in the photoreceptor OS (D) and the number of PCARE puncta per 50 μm^2^ (E). Quantification of GT335 expression was performed as the intensity of GT335 expression in the ONL (F) and the spread of GT335 tubulin bundles in photoreceptor axonemes (G). All data presented as the mean±s.d. (*n*=6 organoids). Significance was determined by Mann–Whitney test or two-tailed unpaired *t*-test (**P=0.0099; ***P=0.0001; ****P<0.0001; ns, not significant).

Previous work has suggested a role for INPP5E in regulating actin in mouse photoreceptors (Gupta et al., 2025). Therefore, we speculated that the same could be true in ROs. We utilized phalloidin to visualize filamentous actin (F-actin) and determine whether INPP5E plays a role in regulating the actin network in developing photoreceptors. In control ROs, phalloidin was expressed at the outer limiting membrane (Fig. 6A); however, small extensions of phalloidin were visible extending into the CC of photoreceptors. In *INPP5E^D477N/D477N^* ROs phalloidin co-localized with the outer limiting membrane, however it extended further into the CC than in control ROs (control, 2.37 μm; *INPP5E^D477N/D477N^*, 3.37 μm) (Fig. 6C).

To gain further insight into the possible dysregulation of the actin protein network, we performed a colocalization experiment with the photoreceptor actin regulator PCARE, which is expressed at the base of the OS and plays an important role in the initiation of new OS disc formation (Corral-Serrano et al., 2020; Spencer et al., 2019). The number of PCARE puncta were increased in *INPP5E^D477N/D477N^* organoids (control, 22.7 puncta per 50 μm^2^; *INPP5E^D477N/D477N^*, 35.8 puncta per 50 μm^2^), as seen previously with ARL13B and RHO. However, the area of the PCARE puncta was decreased in *INPP5E^D477N/D477N^* organoids (0.91 μm^2^) compared to control (1.16 μm^2^) (Fig. 6D,E).

We next sought to determine whether the loss of INPP5E affected photoreceptor microtubules. To do so, we used the polyglutamylated tubulin marker GT335. The number of GT335^+^ photoreceptors increased in *INPP5E^D477N/D477N^* ROs compared to control at d230 (control, 31.8±6.4; *INPP5E^D477N/D477N^*, 42.9±5.7; mean±s.d.), whereas the intensity of GT335 expression in the ONL was slightly, but not significantly, decreased in *INPP5E^D477N/D477N^* compared to control ROs (control: 1394 A.U.; *INPP5E^D477N/D477N^*: 1195 A.U.) (Fig. 6B,F). However, interestingly, the loss of INPP5E led to an altered morphology of GT335^+^ tubulin in the photoreceptors, causing the spread of tubulin bundles in the axoneme, measured by the width of GT335 staining, to decrease compared to control (control, 2.62 μm; *INPP5E^D477N/D477N^*, 1.88 μm) (Fig. 6G).

### Rhodopsin and ARL13B localization are altered in *INPP5E^D477N/D477N^* ROs

**Fig. 7.**
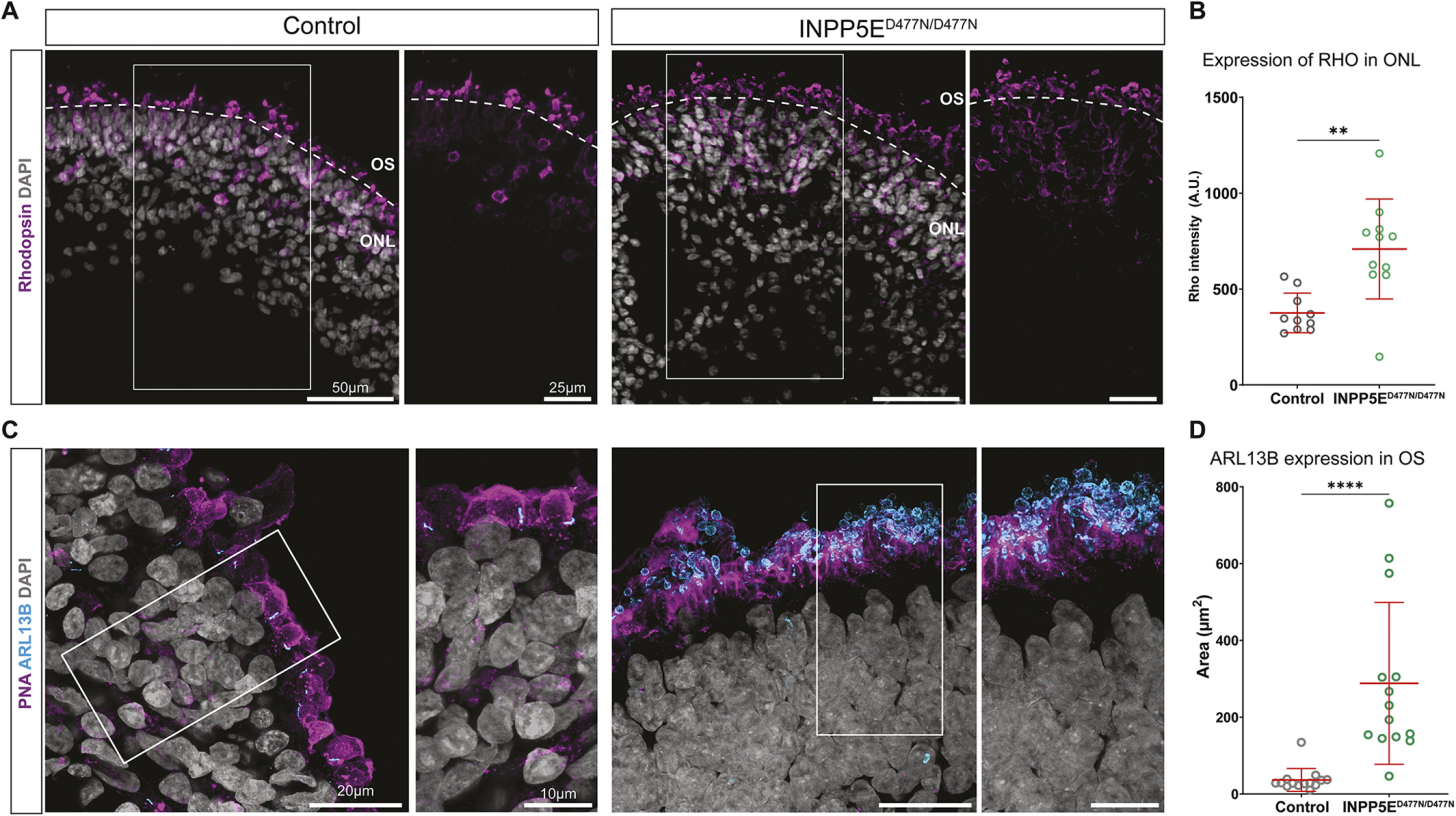
**Rhodopsin and ARL13B trafficking are altered in d230 *INPP5E^D477N/D477N^* organoids.** (A) Representative immunofluorescence of rhodopsin (RHO) and DAPI (gray) in the OS and ONL. The white dashed line shows the separation of the ONL and OS of the ROs. Scale bars: 50 μm and 25 μm. (B) Quantification of expression of RHO in the ONL, measured as the normalized intensity (A.U.). (C) Representative images of ARL13B (blue), PNA (magenta), and DAPI (gray). Scale bars: 50 μm and 25 μm. (D) Quantification of ARL13B expression in PNA^+^ OS, measured as the total area (μm^2^). Data in B and D presented as the mean±s.d. (*n*=6 organoids). Significance was determined by Mann–Whitney test or two-tailed unpaired *t*-test (**P=0.0013; ****P<0.0001).

Previous studies have demonstrated a defect in rhodopsin trafficking in INPP5E mutant mice (Gupta et al., 2025; Sharif et al., 2021), with further studies showing that retention of rhodopsin in the outer nuclear layer of the retina is indicative for several known IRDs (Athanasiou et al., 2025; Gao et al., 2002; Gupta et al., 2025). In line with these studies, immunofluorescence of RHO showed an increase of RHO expression in the ONL of *INPP5E^D477N/D477N^* ROs compared to control (control, 375±103.6 A.U.; *INPP5E^D477N/D477N^*, 708±260.6 A.U.; mean±s.d.) (Fig. 7A,B). We also analyzed opsin R/G localization in the ONL, but saw no change in localization, suggesting that the trafficking defect is rhodopsin specific (Fig. S3).

Interestingly, in *INPP5E^D477N/D477N^* RO we saw an increase of ARL13B expression outside the photoreceptor CC, suggesting a change in the protein localization. To confirm this, we visualized ARL13B with peanut agglutinin (PNA), which stains the OS membrane of photoreceptor cells (Blanks and Johnsonf, 1984) (Fig. 7C). In control organoids, the area of ARL13B expression in the PNA^+^ OS was 36.6 μm^2^, which was significantly increased to 288.2 μm^2^ in *INPP5E^D477N/D477N^* organoids.

## DISCUSSION

Pathogenic variants that cause dysfunction in *INPP5E* lead to both syndromic and non-syndromic RP in humans (Sangermano et al., 2021; Whiting et al., 2024), suggesting an important role for INPP5E in retinal function. Recent studies have used conditional mouse models that selectively knockout INPP5E in the retina or photoreceptors, indicating an important role for INPP5E in photoreceptor OS stability and the maintenance of photoreceptor cells (Gupta et al., 2025; Sharif et al., 2021). However, the role for INPP5E in the human retina remained unknown. Here we used human iPSC-derived ROs to study the role of INPP5E in retinal development and photoreceptor maintenance in a human-based model system. Both control and *INPP5E^D477N/D477N^* organoids differentiated successfully, with no major differences in the general structure of the ROs, suggesting that INPP5E is not crucial for retinal development.

In mature ROs, photoreceptors develop as part of a so-called ‘brush border’ along the outer edge of the organoid, emerging as early as d150 and maturing by d230. These photoreceptors express both rod- and cone-specific proteins including rhodopsin and opsin, respectively. The number of both rod and cone photoreceptors in *INPP5E^D477N/D477N^* ROs were increased compared to control organoids; however, the OS membranes of photoreceptors in *INPP5E^D477N/D477N^* organoids were significantly longer than control ROs. Previously published work using conditional knockout mouse models of INPP5E showed that the loss of INPP5E led to a decrease in OS length due to a decrease in OS disc stability – opposite to what was seen in the *INPP5E^D477N/D477N^* ROs (Gupta et al., 2025; Sharif et al., 2021). The changes observed in RHO- and opsin- positive structures, as well as the affected ARL13B distribution and rhodopsin localization, support the conclusion that INPP5E loss affects photoreceptor OS organization in ROs. However, it is important to note that immunofluorescence analysis lacks the ultrastructural resolution of OS disc organization. Thus, further ultrastructural analysis will be valuable to further define the precise nature of these OS defects.

This opposing result is likely due to the differences in the structure of OSs in *in vivo* mouse models versus the *in vitro* ROs. Typically, OSs are filled with highly organized OS discs, responsible for light capture to initiate phototransduction. Although ROs are able to generate short OSs with rudimentary OS discs, they lack the structure and organization of *in vivo* photoreceptors. This is likely due to the lack of the retinal pigment epithelium (RPE) cell layer, which provides both structural and functional support to the photoreceptor OS (Singh and Nasonkin, 2020).

It has been hypothesized that alongside OS disc stability, INPP5E also plays a role in regulating protein trafficking between the inner and OS (Gupta et al., 2025). Here, mature *INPP5E^D477N/D477N^* organoids had accumulation of rhodopsin in the ONL, a phenotype seen previously in multiple RP-associated models including the INPP5E mouse knockout model (Athanasiou et al., 2025; Gupta et al., 2025), validating this as a phenotypic consequence in humans. Similar to what was seen in the INPP5E mouse model (Gupta et al., 2025), we could demonstrate that INPP5E depletion causes defects in the actin cytoskeleton, but we also identified differences in the expression of the photoreceptor actin regulator PCARE. This matches the known role of INPP5E in actin regulation. The extended CC region that is covered by actin could be indicative of a disrupted balance in actin dynamics, potentially due to enhanced PI(3,4,5)P_3_and PI(4,5)P_2_ levels that are not converted due to absence of INPP5E. Raised levels of PI(3,4,5)P_3_ promote AKT phosphorylation, altering actin dynamics, and raised levels of PI(4,5)P_2_ promotes actin nucleation through interaction with the WAVE complex. A disturbed balance of these processes might underly our observations of the extended OS as well as increased levels but altered size of PCARE puncta. The altered tubulin dynamics could then be a result of the disturbed cytoskeletal processes, which are intertwined at the base of the OSs to facilitate proper OS membrane biogenesis. The fact that somewhat different observations were done in the INPP5E knockout mouse model, where reduced phalloidin staining but no alterations in WAVE complex was observed (Gupta et al., 2025), could indicate that the early imbalance in actin dynamics that we observe in the ROs triggers developmental defects that are expressed in altered stoichiometry in the actin dynamics network at postnatal stages. Although tempting, such hypotheses should be made with caution, as species differences could also be at play.

Furthermore, mature *INPP5E^D477N/D477N^* organoids showed mislocalization of ARL13B, which localizes ectopically to the OSs of *INPP5E^D477N/D477N^* photoreceptors, strengthening the hypothesis that INPP5E regulates protein trafficking within the specialized photoreceptor cilium.

This change in ARL13B localization in *INPP5E^D477N/D477N^* photoreceptors might help explain the elongation of photoreceptor OSs. ARL13B is a regulatory GTPase found at the membrane of the primary cilium, with previous work showing that ARL13B plays a role in regulating ciliary length, where increased ARL13B expression results in ciliary elongation (Lu et al., 2015; Shi et al., 2023). The loss of INPP5E function leading to ARL13B mislocalization could be the cause for changes in membrane biogenesis and thus longer OS in the knockout photoreceptors.

Interestingly, ARL13B mislocalization appears only in later phases of organoid differentiation as ARL13B localization and expression were unaffected in the cilia of d35 *INPP5E^D477N/D477N^* organoids, possibly alluding to a different function of INPP5E during early retinal differentiation. In addition, INPP5E loss seemingly led to a delay in photoreceptor precursor cells in early stage ROs, marked by the decrease in RCVN expression and CRX-expressing cells, both of which were rescued by the time the ROs reached maturation at d230. However, this early differentiation phenotype should be interpreted with caution, as the reduction in CRX-positive nuclei did not reach statistical significance. Therefore, these data suggest altered expression of early photoreceptor markers but do not establish a direct role for INPP5E in early retinal differentiation.

A possible scenario for contribution of INPP5E during early RO development comes from studies of INPP5E function in early brain development. The authors of the study used the same *INPP5E^D477N/D477N^* and isogenic control iPSC lines to generate cerebral organoids, where they demonstrate that the loss of INPP5E leads to an increase in sonic hedgehog (SHH) signaling, thus playing an important role in dorsal ventral patterning during brain development (Schembs et al., 2022). Preliminary data from our laboratory suggests that the loss of INPP5E in ROs leads to an increased ciliary expression of both the SHH transducer smoothened (SMO) and the PI(4,5)P_2_-binding protein TULP3, a negative regulator of SHH signaling (Fig. S4). Although contradictory, a similar mislocalization of both SMO and TULP3 is seen in *INPP5E-*knockout cerebral organoids (Schembs et al., 2022) whereas ciliary TULP3 accumulation is present in the retina of mice lacking *INPP5E* (Sharif et al., 2021).

In the retina, SHH plays a role in regulating cell proliferation and differentiation during retinogenesis – with the activation of SHH leading to faster division of proliferative cells (Locker et al., 2006). Therefore, it is possible that the loss of INPP5E in early RO development leads to an increase in SHH signaling, thus increasing the pool of proliferative cells that would go on to form retinal precursor and photoreceptor cells – explaining the increase in DAPI^+^ cells, the increase of total rod and cone photoreceptor cells in mature ROs, and the increase in PCARE and NPHP1 puncta within these photoreceptors. Although our findings support a role for INPP5E in signaling regulation and/or progenitor dynamics during early RO development, we cannot exclude that altered cell survival, including reduced cell death in *INPP5E^D477N/D477N^* ROs, might contribute to the phenotype, as cell death was not directly assessed in this study. Future work to assess the role of SHH signaling during retinogenesis will help elucidate this hypothesis further.

To conclude, we generated ROs from human iPSCs containing a knockout of *INPP5E*, which we used to provide further insight into INPP5E in both developing and mature photoreceptors, thus building upon previous work performed in mouse models (Gupta et al., 2025; Sharif et al., 2021). Our findings indicate a complex role of INPP5E in the retina, both during early retinal differentiation and in mature photoreceptors, highlighting the importance of INPP5E in regulating protein trafficking through the CC and maintenance of the photoreceptor OS membrane. Furthermore, this study shows that ROs can be a useful model system to study INPP5E-derived retinal defects. It is important to note, though, that ROs are taken out of context of the native organism, leading to structural differences compared to endogenous tissues, for instance the lack of supporting RPE cells and vasculature (Inagaki et al., 2025; Singh and Nasonkin, 2020). Moreover, human ROs model early retinal development, following a similar developmental profile to that of a human fetal retina, limiting the opportunities to use ROs as a model for slow progressing retinal degeneration (Cui et al., 2020; Sridhar et al., 2020). Although continued work is required to optimize ROs to accurately model retinal degeneration, our data indicates their suitability for future studies towards therapeutic approaches to rescue of the phenotype caused by the loss of INPP5E.

## MATERIALS AND METHODS

### iPSC-derived RO differentiation

Human induced pluripotent stem cells (iPSCs) were cultured on Matrigel (Corning, 734-0269)-coated six-well plates in mTesR1 medium (STEMCELL Technologies, 85850) containing 1% penicillin-streptomycin (Pen/Strep; Sigma-Aldrich, P4333). Cells were passaged with 0.5 mM EDTA (Invitrogen, 11568896) and maintained at 37°C with 5% CO_2_. CRISPR/Cas9-generated INPP5E knockout iPSCs, containing a homozygous point mutation (D477N/D477N, *INPP5E^D477N/D477N^*) and their corresponding isogenic control line were characterized previously (Schembs et al., 2022).

iPSCs were differentiated into ROs using a previously established protocol (Georgiou et al., 2020; Kuwahara et al., 2015; Whiting et al., 2026). Briefly, iPSCs at 75–80% confluency was dissociated into single cells using Accutase (Gibco, A1110501) and seeded at 7000 cells per well in ultra-low adhesion U-Bottom 96-well plates (Corning, 7007) in mTesR1 medium containing 10 μM Y-27632 (a ROCK inhibitor; Sigma-Aldrich, Y0503). After 48 h (day 0 of differentiation), 200 μm differentiation medium [41% IMDM (Sigma, I3390), 41% HAM's F12 (Sigma, N6658), 15% KOSR (Gibco, 10828028) 1% GlutaMAX (Gibco 35050061), 1% lipids (Gibco, 11905031), 1% Pen/Strep, and 0.2% 1-Thioglycerol (Sigma-Aldrich, M6145)] was added to each well. Every other day, organoids were subjected to half medium changes, and on day 6 of differentiations a 2.25 nM BMP4 (Sigma, SRP3016) pulse was added. At d18, ROs were moved to ultra-low adhesion six-well plates (Corning, 3471), and the medium was changed to maintenance medium containing 9-cis-retinol [DMEM/F12 (Gibco, 31330038), 10% FBS, 1% N2 (Gibco, 17502048), 1% Pen/Strep, 1% GlutaMAX, 0.1 mM taurine (Sigma Aldrich, T0625), 0.25 µg/ml Amphotericin B (Gibco, 15290018), and 0.5 μM 9-cis-Retinol (Sigma-Aldrich, R5754)]. Medium was changed every 3–4 days, and 9-cis-retinol was removed after 120 days of differentiation.

Brightfield images of RO differentiation were captured at multiple timepoints throughout the differentiation using the EVOS XL Core Cell Imaging System (Invitrogen).

### Immunofluorescence

Two differentiations were completed per cell line and 3–4 ROs were collected at day 35 (d35) and day 230 (d230) per differentiation. ROs were washed in PBS and fixed in 4% PFA at room temperature (RT) for 20 min and cryoprotected in 30% sucrose overnight at 4°C. ROs were then embedded in OCT compound (Sakura Finetek Europe, 25322-68-3), and slowly frozen on dry ice. Cryosections were collected at 12 μm thick and stored at −20°C until used for immunofluorescence.

In short, sections were washed briefly in PBS to remove OCT and incubated in blocking buffer [0.3% Triton-X and 2.5% natural goat serum (NGS) in PBS] for 1 h at RT. Slides were incubated overnight at 4°C in primary antibody solution, diluted in antibody dilutant (0.1% Triton-X, 0.5% NGS in PBS), washed in PBS, and incubated at RT for 1 h in secondary antibody mixes diluted in PBS before mounting with Fluoromount-G. Antibodies used in this study are listed in Table S1. Images were captured with a 63× oil immersion objective on either the LSM-900 laser scanning microscope with Airyscan (Zeiss) or Axio Imager Z2 fluorescence microscope equipped with Apotome (Zeiss). Final images are represented as maximum projection of *Z*-stacks.

### Protein lysis of ROs

For bulk proteomic analysis of ROs through development, 8–12 control and *INPP5E^D477N/D477N^* organoids were collected at d0 (undifferentiated iPSCs), d35 (early RO development) and d230 (mature ROs). Organoids were washed twice in PBS and homogenized using the TissueLyser III (Qiagen) and lysed in RIPA buffer [50 mM Tris-HCl pH 7.5, 150 mM NaCl, 1% NP-40, 0.1% SDS (Life Technologies, 15553-027), 0.5% sodium deoxycholate, 1 mM UltraPure EDTA pH 8.0 (Invitrogen, 11568896)] containing cOmplete protease inhibitor cocktail (Roche, 11836170001). Samples were centrifuged for 15 mins at 14,000 ***g*** at 4°C. Protein concentration of the lysates was measured using the Pierce BCA protein assay (Life Technologies, 23225), and two technical replicates of 15 μg aliquots were snap frozen and stored at −80°C until mass spectrometry analysis.

### Liquid chromatography-mass spectrometry analysis and data analysis

Proteomic analysis was performed on RO samples as described previously (Mattern et al., 2025; Merk et al., 2024). Briefly, tryptic peptides were processed for LC-MS/MS using data- independent acquisition mode (DIA) on the QExactive Plusmass spectrometer (Thermo Fisher Scientific). Raw data files were analyzed using DIA-NN 1.8.2 (Demichev et al., 2019) in library-free mode against the human database (Swiss-Prot, May 2023, #20,401 proteins), and high-accuracy spectra with a minimum false discovery rate (FDR) of 0.01 and tryptic peptides were used for protein abundance label free quantification.

Statistical analysis was calculated using Perseus (version 1.6.15.0) (Tyanova et al., 2016). Significant proteins were identified using a two-tailed unpaired Student's *t*-test with permutation-based FDR of <0.05 and a Significance A (SignA) outlier test with Benjamini–Hochberg FDR correction of <0.05. Gene Ontology (GO) analysis was performed on significant proteins using the Gene Ontology Resource (Aleksander et al., 2023; Ashburner et al., 2000; Thomas et al., 2021) for biological processes (BP), molecular functions (MF) and cellular components. Enriched terms were filtered using a Fisher's Exact test and corrected using the Bonferroni correction for multiple testing.

### Quantification and statistical analysis

Immunofluorescence images were quantified using Fiji software (Schindelin et al., 2012). For each timepoint analyzed, three to four organoids over two differentiations were analyzed. At least two images were taken per organoid per timepoint, and quantification was performed manually for area of expression, mean fluorescence intensity (MFI), length or frequency of the relevant antibody staining.

Specifically, ONL length was measured manually using antibodies against phalloidin and DAPI to identify the ONL (as shown in Fig. S5). To quantify nuclei count in the ONL, two 50 μm^2^ areas were defined per image and the total number of DAPI^+^ nuclei were counted in the defined areas. CRX percentage was calculated as the percentage of nuclei positive for CRX relative to the total number of DAPI^+^ nuclei within the ONL region. RCVN expression was measured as the total area of RCVN-positive staining within the identified ONL.

Quantification of cilia and cilia-associated proteins was completed using antibodies against ARL13B, NPHP1, PCNT, TULP3, SMO and PCARE as the total number of cilia or puncta within two 50 μm^2^ regions defined per image. The length of ARL13B^+^ cilia, phalloidin within the CC, and width of CT335 tubules were measured manually. To quantify expression of INPP5E within the cilium, masks of ARL13B^+^ cilia were generated and the MFI of INPP5E was measured and normalized to background INPP5E staining.

Finally, quantification of photoreceptor morphology was performed as the total number of Opsin-R/G and Rhodopsin positive photoreceptors per μm of RO, and cone/rod ratio was determined as a percent of cone and rod photoreceptors per image. OS length was measured manually. Trafficking of RHO, Opsin-R/G, and ARL13B was measured as the MFI of the protein of interests within the ONL (RHO and opsin) or PNA (ARL13B) of d230 ROs.

Statistical analyses were performed using GraphPad Prism 10 (GraphPad, San Diego, CA, USA). Comparison between groups was performed using either a two-tailed unpaired *t*-test, one-way ANOVA with post-hoc Tukey's test or Mann–Whitney test, as indicated. Significance between groups was determined if *P*-values were less than 0.05, and significance is indicated with asterisks (*P=0.05–0.01; **P=0.01–0.001; ***P=0.001–0.0001; ****P<0.0001).

## Supplementary Material



10.1242/joces.264431_sup1 Supplementary information

Table S2. Retinal organoid proteomics results.
