# Supplementary material for: Loss of INPP5E affects photoreceptor outer segment membrane biogenesis in iPSC-derived human retinal organoids
Source: J Cell Sci. 2026 Jul 21;139(14):jcs264431. doi: 10.1242/jcs.264431 (PMC13450897; doi:10.1242/jcs.264431)
Supplement: Supplementary file 1 [file joces-139-264431-s1.pdf]

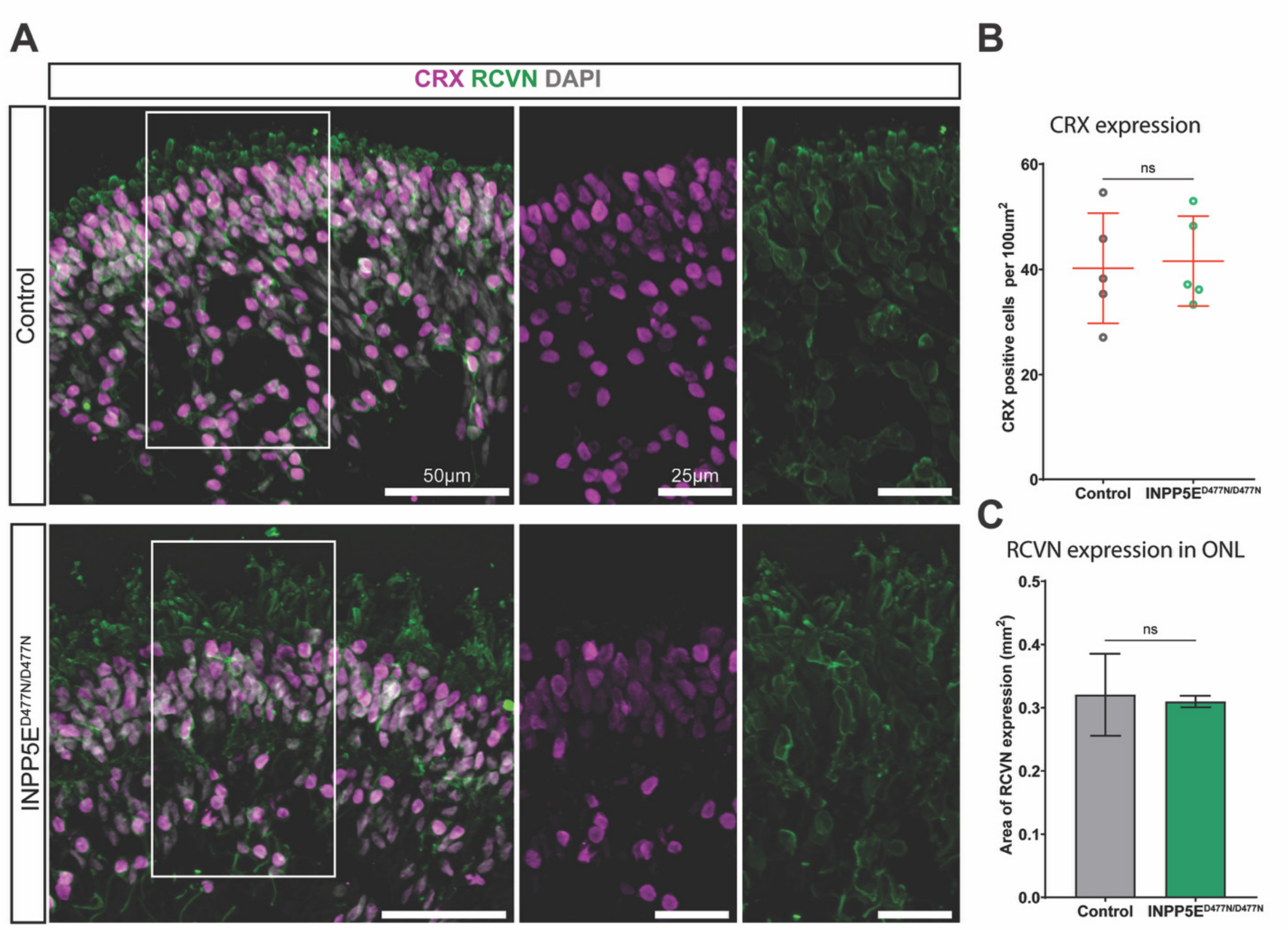

Fig. S1. Expression of CRX and RCVN is unaffected in d230 ROs. (A) Representative images of retinal precursor markers CRX (magenta) and RCVN (green) in d230 retinal organoids (Scale bar: 50µm and 25µm). (B-C) Quantification of the retinal precursor markers as the percent CRX positive nuclei (B) and the area of RCVN expression in the ONL (C) (n=4 organoids).

Journal of Cell Science • Supplementary information

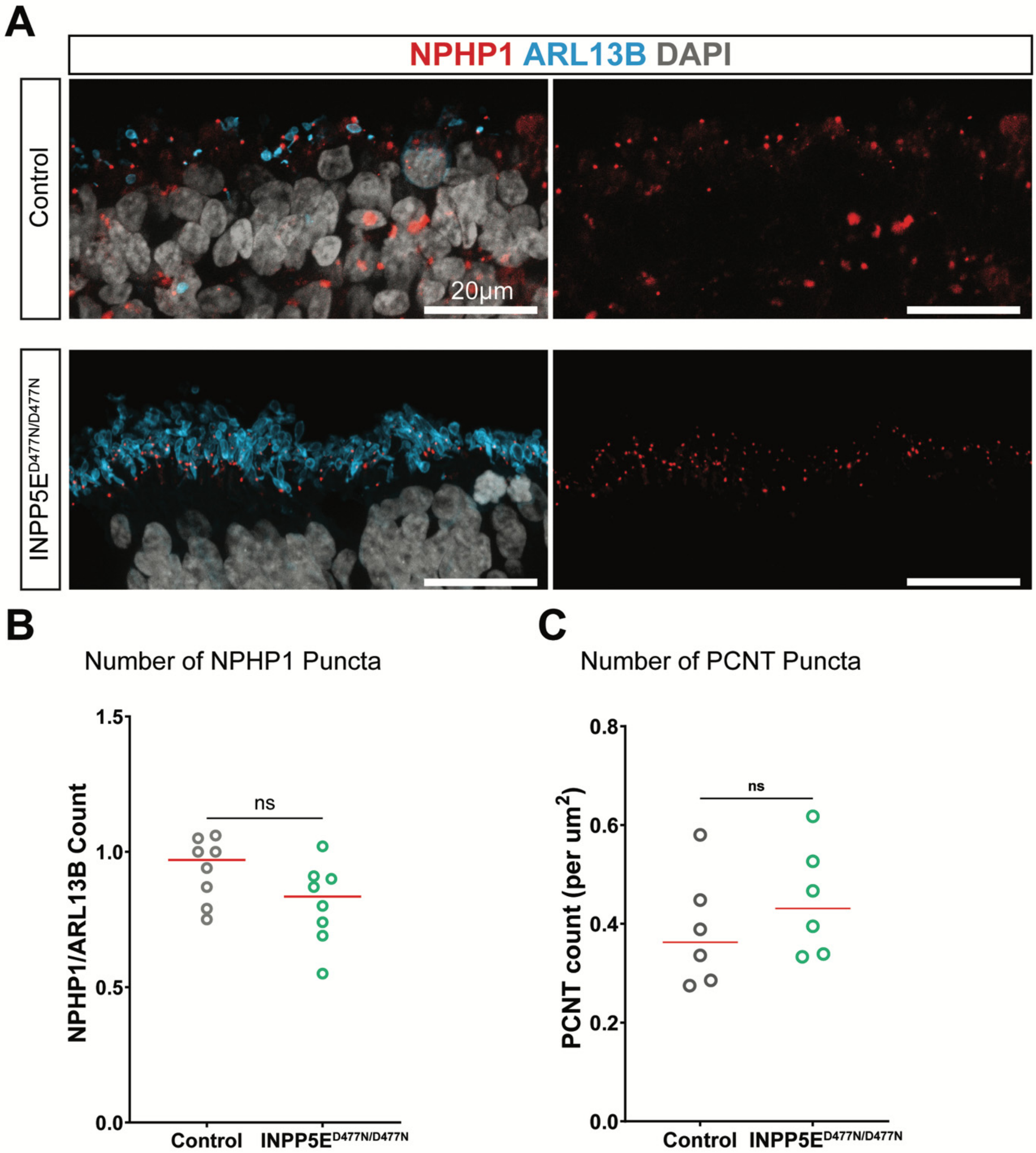

Fig. S2. Basal body and transition zone markers are not affected in INPP5E$^{D477N/D477N}$ organoids. (A) Representative images of d230 ROs stained for NPHP1 (red), ARL13B (blue), and DAPI (grey). (B) Quantification of NPHP1 puncta as the count per ARL13B^{+} cilia. (C) Pericentrin (PCNT) puncta count as the number of PCNT puncta per µm^2 (n=6 organoids).

Journal of Cell Science • Supplementary information

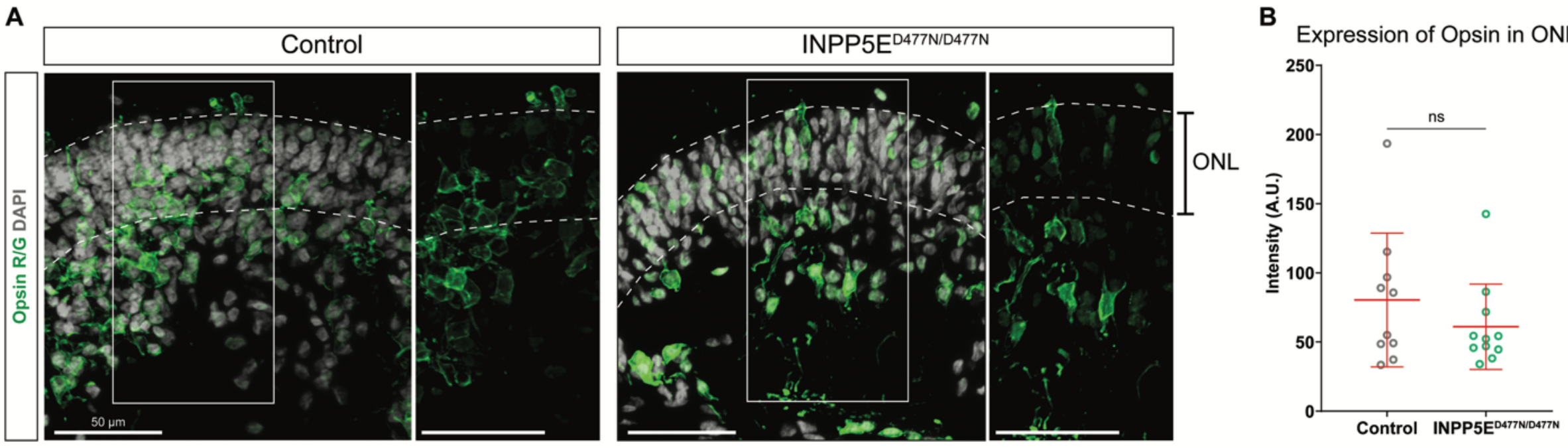

Fig. S3. Opsin localization is not altered in INPP5E$^{D477N/D477N}$ ROs. (A) Representative images of Control and INPP5E$^{D477N/D477N}$ ROs stained for Opsin R/G (green) and DAPI (grey). Dashed lines represent the boundary of the ONL within the ROs (scale: 50 µm) (B) Quantification of Opsin expression in the ONL was measured as normalized intensity (A.U.) (n=6 organoids).

Journal of Cell Science • Supplementary information

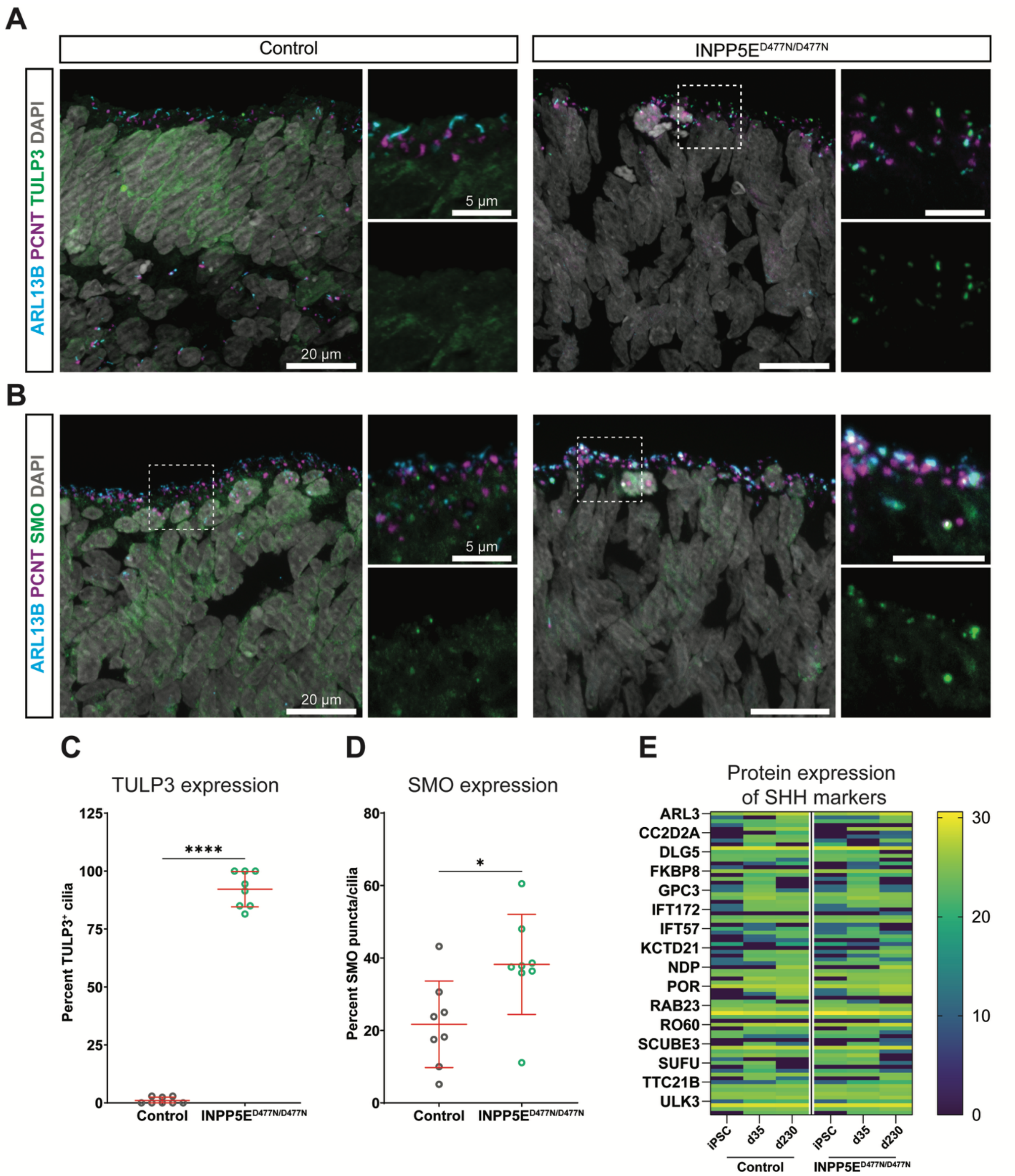

Fig. S4. Localization of SHH regulators are affected in d35 INPP5E$^{D477N/D477N}$ organoids. (A-B) Representative images of d35 retinal organoids stained with antibodies against ARL13B (blue), PCNT (magenta), and TULP3 (green, A) or SMO (green, B) (Scale: 20 µm and 5 µm). Quantification of TULP3 and SMO localization was measured as the percent TULP3^{+} cilia (C) or the percent SMO^{+} cilia (D), presented as the mean±s.d (n=2 organoids). (E) Protein expression of SHH markers of iPSC, d35, and d230 retinal organoids identified in the bulk proteomic analysis, as identified by GO term analysis (GO:0007224). Significance was determined by an unpaired t-test (* $p = 0.0229$, **** $p < 0.0001$).

Journal of Cell Science • Supplementary information

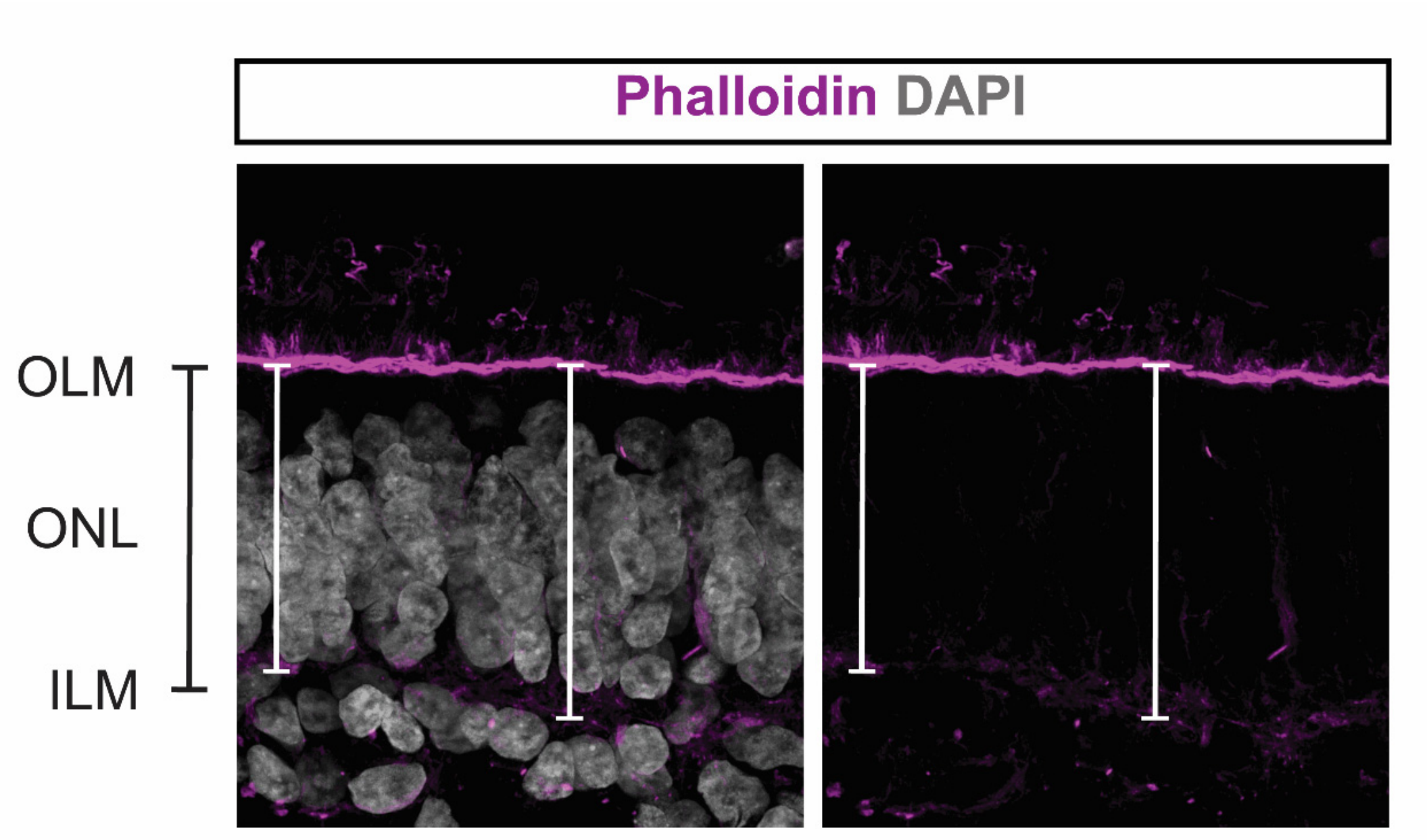

Fig. S5. Method of outer nuclear layer length measurement. The ONL can be identified by the densely packed nuclei, visualized using DAPI, or using phalloidin to visualize the outer limiting membrane (OLM) and inner limiting membrane (ILM). Manual length measurement of the ONL was completed at two separate areas per image.

Table S1. Antibodies Table

Antibody	Source	Identifier	Dilution
Mouse anti-CRX	Abnova	H00001406-M02	1:200
Mouse anti-ARL13B (IgG2a)	NeuroMab	75-287	1:500
Rabbit anti-ARL13B	Proteintech	17711-1-AP	1:500
Rabbit anti-Opsin R/G	Millipore	AB5405	1:200
Mouse anti-Rhodopsin	Novus Biologicals	NBP2-59690-25ug	1:500
Rabbit anti-RCVN	Chemicon	AB5585	1:1000
Mouse anti-PCNT (IgG1)	Abcam	AB28144	1:500
Rabbit anti-INPP5E	Proteintech	17797-1-AP	1:500
Rabbit anti-PCARE	Homemade	N/A	1:250
Mouse anti-GT335	AdipoGen Life Sciences	AG-20B-0020-C100	1:250
Mouse anti-NPHP1	Bernhard Schermer Lab	N/A	1:500
Rabbit anti-TULP3	Proteintech	13637-1-AP	1:250
Mouse anti-SMO (IgG2a)	Santa Cruz Biotechnology	Sc- 166685	1:250
Anti- Lectin PNA (568 conjugate)	Thermo Fisher Scientific	L32458	1:500
Anti-Phalloidin (568 conjugate)	Invitrogen	A12380	1:250
Goat anti-Mouse IgG1 (647)	Invitrogen	10789384	1:500
Goat anti-Mouse IgG1 (488)	Invitrogen	A21121	1:500
Goat anti-Mouse IgG2a (647)	Thermo Fisher Scientific	A21241	1:500
Goat anti-Mouse IgG2a (488)	Thermo Fisher Scientific	A21131	1:500
Goat anti-Mouse IgG2a (568)	Invitrogen	A21134	1:500
Goat anti-Mouse (488)	Invitrogen	A11029	1:500
Goat anti-Mouse (568)	Life Technologies	A11031	1:500
Goat anti-Rabbit (647)	Invitrogen	A21245	1:500
Goat anti-Rabbit (488)	Molecular Probes	A11008	1:500
Goat anti-Rabbit (568)	Molecular Probes	A11011	1:500
DAPI	Thermo Fisher Scientific	D1306	1:100

Table S2. Retinal organoid proteomics results.

Available for download at
https://journals.biologists.com/jcs/article-lookup/doi/10.1242/jcs.264431#supplementary-data
