# Supplementary material for: Loss of INPP5E affects photoreceptor outer segment membrane biogenesis in iPSC-derived human retinal organoids
Source: J Cell Sci. 2026 Jul 21;139(14):jcs264431. doi: 10.1242/jcs.264431 (PMC13450897; doi:10.1242/jcs.264431)
Supplement: Supplementary file 2 [file joces-139-264431_review_history.pdf]

Loss of INPP5E affects photoreceptor outer segment membrane biogenesis in iPSC-derived human retinal organoids

Kae R. Whiting, Mariam G. Aslanyan, Lynn van Summeren, Tina Beyer, Katrin Dahlke, Thomas Theil, Karsten Boldt and Ronald Roepman
DOI: 10.1242/jcs.264431

Editor: Pleasantine Mill

Review timeline

Original submission:	5 September 2025
Editorial decision:	8 November 2025
First revision received:	6 March 2026
Editorial decision:	4 May 2026
Second revision received:	9 June 2026
Accepted:	11 June 2026

Original submission

First decision letter

MS ID#: jcs.264431

MS TITLE: Loss of INPP5E affects photoreceptor outer segment membrane biogenesis in iPSC-derived human retinal organoids

AUTHORS: Kae R Whiting; Mariam G Aslanyan; Tina Beyer; Katrin Dahlke; Karsten Boldt; Ronald Roepman
ARTICLE TYPE: Research Article

Dear Kae and Ronald,

We have now reached a decision on the above manuscript.

As you will see, the reviewers gave favourable reports but raised some critical points that will require amendments to your manuscript. I hope that you will be able to carry these out because I would like to be able to accept your paper, depending on further comments from reviewers.

Reviewer 1

Whiting et al in this manuscript describe the role of INPP5E in retinal development and photoreceptor maintenance. INPP5E is a known gene that causes retinal ciliopathies. The authors used INPP5E CRISPR-engineered functional knockout iPSCs to differentiate them into retinal organoids in order to study the retinal phenotype that could cause the absence of the functional protein, aiming to define its role in retinal development.

This is the first study that implies the generation of INPP5E knockout retinal organoids and it is really interesting in the field of retinal ciliopathies. It is very well organised, with different techniques to analyse the phenotype in early stage and late stage retinal organoids.

There are some comments that the authors need to address:

© 2026. Published by The Company of Biologists under the terms of the Creative Commons Attribution License (https://creativecommons.org/licenses/by/4.0/).

-The authors performed proteomic analysis and subsequent gene ontology analysis. However, they do not show the proteins altered that could explain the phenotype in a molecular level. In the discussion they mention the possible alteration of the SHH signalling pathway. Are there proteins implicated in this pathway altered in these organoids? Could they confirm these alterations via western blotting? Are there the same alterations in the late vs the early stage organoids?

-Can the authors present as a supplementary material the dataset from the proteomic analysis?

-Have the authors check if there is any ciliary phenotype in the iPSCs? Do these cells have the same pathways altered in the proteomic analysis?

-Please check the order of the figures. In the figures section, the figure of the proteomic is Fig 2 instead of Fig 3.

Reviewer 2

SUMMARY OF THE ADVANCE MADE IN THIS PAPER AND ITS POTENTIAL SIGNIFICANCE TO THE FIELD

The study by Whiting et al. employs an iPSC line carrying an INPP5E mutation to investigate the role of this enzyme in retinal development and function. The relevance of INPP5E to retinal biology is well established through prior studies linking its mutations to Joubert syndrome and non-syndromic retinitis pigmentosa (RP), conditions typically associated with rod photoreceptor dysfunction, as well as through supporting animal models. Using iPSC-derived retinal organoids, the authors conclude that (1) INPP5E is essential for early retinal development and photoreceptor progenitor differentiation, (2) ARL13B localization, a ciliary marker linked to Joubert syndrome, is disrupted, and (3) outer segment membranes are abnormally elongated in both cones and rods, suggesting structural defects in the absence or catalytic loss of INPP5E. However, these conclusions are not well supported by the experimental evidence presented.

SUGGESTIONS TO AUTHORS

Major comments

I offer the following suggestions/critiques to improve our understanding of INPP5E in iPSC-based RO systems.

1. The authors repeatedly emphasize the importance of studying retinal diseases using iPSC-based systems. While this is a valid point, they also argue that mouse models are unsuitable because they lack a fovea. However, the retinal organoid (RO) system itself also lacks a foveal structure. How, then, does the iPSC-RO system described here for INPP5E accurately recapitulate the patient phenotype, whether Joubert syndrome or RP? This issue is not discussed. Furthermore, as the authors note, the organoid system lacks supporting cell types, such as RPE, and does not exhibit degeneration as observed in animal models. While each experimental system offers unique advantages, a balanced discussion comparing disease phenotypes observed in patients, animal models, and iPSC-derived systems is warranted and is currently missing.

2. The assertion that the mutation renders INPP5E non-functional is based on reference 18; however, this source does not directly demonstrate catalytic inactivity. Instead, it cites earlier work from Christina Mitchell's group on a protein referred to as 72-5ptase. The authors should clarify the equivalence between 72-5ptase and INPP5E, as their interpretation of loss of function depends entirely on this connection. Additionally, prior studies from Wolfgang Baehr's group have shown that INPP5E localizes to the Golgi apparatus in the retina. The manuscript does not discuss or evaluate whether the reported phenotype could be linked to potential Golgi dysfunction, which should be addressed.

3. INPP5E deficiency is commonly evidenced by mislocalization of PIP-binding proteins such as TULP1 (also associated with RP in humans) or TULP3. Did the authors examine or observe any changes in the localization of these proteins?

© 2026. Published by The Company of Biologists under the terms of the Creative Commons Attribution License (https://creativecommons.org/licenses/by/4.0/).

4. Figure 1 The primary data (images) used to derive ONL length and cell counts are missing. The methods for ONL measurement and quantification are unclear: how was the ONL delineated, labeled, and counted? What controls were used in these analyses? Were nuclei in other retinal layers, such as the INL, also quantified to assess specificity? Additionally, were differences observed between rod and cone nuclei? Finally, if validated, how do the authors explain the apparent increase in ONL counts observed in the absence of INPP5E?

5. Figure 2 (labeled as Fig 3) The figure is titled "Delayed expression of photoreceptor precursor markers." Data are shown for day 35; however, at day 230, are recoverin and CRX staining patterns comparable between normal and mutant retinal organoids? CRX-positive cells were not significantly altered at day 35 (panel B). The calculation of CRX-positive cell percentages (Line 99) is unclear—does this represent the proportion of photoreceptor precursors that are CRX positive? How were photoreceptor precursors identified or estimated? Additionally, standard deviations (SD) are not shown.

6. Figure 3(labeled as Fig 2). Did the authors observe any significant changes in INPP5E expression in the proteomics study? Additionally, the GO terms cell projection organization and extracellular matrix are mentioned, but their relevance to INPP5E function is not discussed. How do these pathways relate to the specific mutation studied?

7. Figure 4, Structural changes in cilia are assessed using ARL13B labeling, a marker known to interact with INPP5E. To validate these findings, the authors should include additional photoreceptor ciliary markers such as acetylated tubulin, RPGR, or RP1, as PCNT does not reliably indicate ciliary morphology (lines 132-133). A table summarizing cilia number and length measurements at d35 and d230 would help readers better visualize the observed changes.

8. Lines 128-129: The reported values (2627 ± 1669) are unclear, do these represent colocalization coefficients (ARL13B with INPP5E)? Line 131: A semicolon is missing after "d230." The variability in the data (358 ± 496) is unusually high, are the authors certain that INPP5E is being correctly detected in the mutant retinal organoids.

9. Figure 5 titled "Photoreceptor morphology is altered in INPP5E…, this figure presents a key conclusion of the study but lacks sufficient supporting evidence. Rhodopsin appears similarly mislocalized in both control and mutant retinal organoids, yet the authors attribute this mislocalization specifically to the mutation. Could the apparent increase in opsin-positive cells instead reflect the changes in ONL thickness noted in Figure 1? Furthermore, the claim regarding altered outer segment (OS) length requires validation by transmission electron microscopy to substantiate the morphological findings.

10. Figure 6, The interpretation of changes in GT335 width is unclear. What does this alteration signify regarding photoreceptor function or morphology? The authors should clarify why INPP5E deficiency would be expected to influence this parameter and how it relates to ciliary or outer segment structure.

11. Figure 7: No clear evidence of altered opsin trafficking is apparent. Even if slight differences exist in the mutant line, these are likely indirect effects. Have the authors examined other outer segment markers beyond opsin to strengthen this conclusion? Importantly, ARL13B does not colocalize with opsin, indicating that ARL13B is not localized to the outer segment. The apparent increase in ARL13B-positive vesiculation suggests possible membrane trafficking abnormalities, which should be further examined by higher-resolution TEM imaging.

12. Supplementary Fig 1, rather than NPHP1, a basal body marker, the authors should include additional ciliary markers as recommended earlier to provide a more comprehensive assessment of ciliary structure.

Minor comments

Figures 2 and 3 are mislabeled. Fig. 3 should be Fig. 2 and vice versa.

© 2026. Published by The Company of Biologists under the terms of the Creative Commons Attribution License (https://creativecommons.org/licenses/by/4.0/).

Authors repeatedly use the term "outer segment stability." What is meant by stability of OS? This term needs to be defined. Sometimes this is referred to as "OS disc stability."

First revision

Author response to reviewers' comments

Point-by-point responses to the Reviewers' comments

First of all, we would like to thank the Editor and the reviewers for their constructive feedback and appreciate their comments and suggestions. These gave us the chance to improve our manuscript, include critical data and improve our discussion. All these points are addressed below.

Comments from the Reviewers:

Reviewer 1:

Whiting et al in this manuscript describe the role of INPP5E in retinal development and photoreceptor maintenance. INPP5E is a known gene that causes retinal ciliopathies. The authors used INPP5E CRISPR-engineered functional knockout iPSCs to differentiate them into retinal organoids in order to study the retinal phenotype that could cause the absence of the functional protein, aiming to define its role in retinal development.

This is the first study that implies the generation of INPP5E knockout retinal organoids and it is really interesting in the field of retinal ciliopathies. It is very well organised, with different techniques to analyse the phenotype in early stage and late-stage retinal organoids.

There are some comments that the authors need to address:

- The authors performed proteomic analysis and subsequent gene ontology analysis. However, they do not show the proteins altered that could explain the phenotype in a molecular level. In the discussion they mention the possible alteration of the SHH signalling pathway. Are there proteins implicated in this pathway altered in these organoids?

We thank the reviewer for these important points. Our bulk proteomic analysis identified several thousand significantly enriched proteins across differentiation stages (d0, d35, d230), confirming robust retinal fate acquisition (Fig. 3). We systematically interrogated these datasets to identify molecular candidates that could mechanistically explain the observed phenotypes, including targeted analysis of cilia-associated and SHH pathway-related proteins.

Although SHH pathway components were detected in the dataset, we did not observe a statistically robust or internally consistent enrichment pattern that would allow us to conclusively attribute the retinal phenotype to differential abundance of canonical SHH pathway proteins at the bulk proteomic level. This is likely due to (i) the cell-type heterogeneity of the organoids, and (ii) the fact that SHH pathway regulation is frequently mediated by subcellular localization and post-translational mechanisms rather than strong changes in total protein abundance.

To further explore potential SHH pathway alterations beyond bulk abundance measurements, we performed antibody-based immunofluorescence analyses in early (d35) and mature (d230) retinal organoids, focusing on the SHH transducer SMO and the $PI(4,5)P_2$-binding protein TULP3. These proteins were selected based on prior reports demonstrating altered ciliary localization in INPP5E- deficient systems. In d35 organoids, we observed increased ciliary localization of both SMO and TULP3 in $INPP5E^{KD}$ organoids, consistent with findings reported in INPP5E-deficient cerebral organoids and mouse retina models (Schembs, L. (2022) DOI: 10.1016/j.celrep.2022.110811; Sharif, A.S. (2021) DOI: 10.1016/j.jbc.2021.100529) and coinciding with the developmental delay in photoreceptor precursor specification described in Figure 2. This is consistent with a potential early developmental contribution of SHH signaling.

© 2026. Published by The Company of Biologists under the terms of the Creative Commons Attribution License (https://creativecommons.org/licenses/by/4.0/).

We have now included a supplementary figure (Fig. S4) summarizing these new results, including the expression levels of SHH-related proteins identified in the proteomic dataset (panel E), and more explicitly report these findings in the revised manuscript.

- Could they confirm these alterations via western blotting?

Given that the observed alterations are subcellular and cilium-specific, western blotting of whole organoid lysates would likely not provide sufficient spatial resolution to validate this phenotype and may obscure subtle but biologically relevant trafficking changes. For this reason, we prioritized high- resolution immunofluorescence-based analysis.

- Are there the same alterations in the late vs the early-stage organoids?

Attempts were made to extend these analyses to d230 organoids; however, due to technical imaging limitations and limited tissue availability from independent differentiations, the late-stage results were inconclusive. At present, we therefore cannot draw firm conclusions regarding stage-specific SHH pathway dynamics. Our data do suggest that if SHH pathway alterations occur, they are more likely to play a role during early retinal differentiation rather than in mature photoreceptor maintenance. Definitive assessment of stage-dependent SHH pathway regulation will require additional independent differentiations and quantitatively powered analyses, which are beyond the scope of the current study but represent an important direction for future work.

Given these restrictions, we have presented these data as preliminary (Fig. S4) and have carefully moderated our interpretation in the Discussion (lines 270-276).

- Can the authors present as a supplementary material the dataset from the proteomic analysis?

A supplemental file containing both the raw proteomic data and the processed datasets used in this analysis has been added as a supplemental file.

- Have the authors check if there is any ciliary phenotype in the iPSCs? Do these cells have the same pathways altered in the proteomic analysis?

We did not assess the ciliary phenotype or pathway alterations at the iPSC stage. This choice was made for conceptual and technical reasons:

First, the primary objective of our study is to interrogate disease mechanisms in the context of human retinal biology. Accordingly, all phenotypic and proteomic analyses were performed in differentiated retinal organoids, which more faithfully recapitulate retinal architecture, cell-type composition, and the specialized ciliary biology of photoreceptors than undifferentiated iPSCs. While analysis at the iPSC stage could provide insight into general stem cell biology, it is unlikely to yield retina-relevant mechanistic information beyond what is obtained in the organoid system, which represents the disease-relevant tissue context.

Second, iPSCs are highly proliferative and asynchronous in the cell cycle, with a relatively low basal ciliation rate (approximately 20-50% under standard conditions; Haenseler, W. (2025) DOI: 10.3389/fcell.2025.1516596). Experimental induction of ciliation through serum starvation or confluency can promote spontaneous differentiation and compromise pluripotency, thereby introducing significant confounding variables into comparative ciliary analyses. These technical limitations reduce the interpretability of ciliary phenotyping in iPSCs, particularly in the context of a study focused on tissue-specific disease mechanisms.

- Please check the order of the figures. In the figures section, the figure of the proteomic is Fig 2 instead of Fig 3.

We apologize for this error in the order of the figures, which has been checked and adjusted accordingly.

Reviewer 2:

© 2026. Published by The Company of Biologists under the terms of the Creative Commons Attribution License (https://creativecommons.org/licenses/by/4.0/).

SUMMARY OF THE ADVANCE MADE IN THIS PAPER AND ITS POTENTIAL SIGNIFICANCE TO THE FIELD.

The study by Whiting et al. employs an iPSC line carrying an INPP5E mutation to investigate the role of this enzyme in retinal development and function. The relevance of INPP5E to retinal biology is well established through prior studies linking its mutations to Joubert syndrome and non-syndromic retinitis pigmentosa (RP), conditions typically associated with rod photoreceptor dysfunction, as well as through supporting animal models. Using iPSC-derived retinal organoids, the authors conclude that (1) INPP5E is essential for early retinal development and photoreceptor progenitor differentiation, (2) ARL13B localization, a ciliary marker linked to Joubert syndrome, is disrupted, and (3) outer segment membranes are abnormally elongated in both cones and rods, suggesting structural defects in the absence or catalytic loss of INPP5E. However, these conclusions are not well supported by the experimental evidence presented.

SUGGESTIONS TO AUTHORS

Major comments

I offer the following suggestions/critiques to improve our understanding of INPP5E in iPSC-based RO systems.

1. The authors repeatedly emphasize the importance of studying retinal diseases using iPSC-based systems. While this is a valid point, they also argue that mouse models are unsuitable because they lack a fovea. However, the retinal organoid (RO) system itself also lacks a foveal structure. How, then, does the iPSC-RO system described here for INPP5E accurately recapitulate the patient phenotype, whether Joubert syndrome or RP? This issue is not discussed. Furthermore, as the authors note, the organoid system lacks supporting cell types, such as RPE, and does not exhibit degeneration as observed in animal models. While each experimental system offers unique advantages, a balanced discussion comparing disease phenotypes observed in patients, animal models, and iPSC-derived systems is warranted and is currently missing.

We thank the reviewer for the comment and agree that it is important to clarify the differences in model systems to explain why we used iPSC-derived retinal organoids for our study. In lines 61-64, we briefly describe known differences in the structure and function of the mouse retina that can lead to difficulties in modelling retinal disorders *in vivo*, including the lack of a fovea in mice. While retinal organoids don't contain a typical foveal structure, previous research has shown that the cone photoreceptors in retinal organoids can respond to light in a similar manner to the fovea of primates (Saha, A. (2022) DOI: 10.1016/j.stem.2022.01.002).

Differences in the retinal organoid structure and organization compared to the *in vivo* human or mouse eye, as discussed with regard to the lack of supporting RPE cells (line 226-228), can indeed lead to difficulties recapitulating the patient phenotype exactly. Retinal organoids model early retinal development typical for fetal eye development. Patients with variants in *INPP5E* typically have late onset retinal degeneration, making a comparison to patient phenotypes difficult in our model system. However, as the knock-out of *INPP5E* led to a clear phenotype within the retinal organoids, we believe the results are informative for INPP5E function in the retina, even if the phenotype is not directly relatable to patient phenotypes.

To address this concern, we have included a discussion of the limitations of using retinal organoids as a model system to model retinal degeneration in the conclusion of the paper (lines 292-300).

Finally, while we agree that comparing the advantages and disadvantages of both *in vitro* and *in* vivo model systems and compare the phenotypes observed to those of patients with pathogenic variants in *INPP5E*, this is a nuanced discussion that warrants an in depth review of the matter, which we consider beyond the scope of this paper.

2. The assertion that the mutation renders INPP5E non-functional is based on reference 18; however, this source does not directly demonstrate catalytic inactivity. Instead, it cites earlier work from Christina Mitchell's group on a protein referred to as 72-5ptase. The authors should clarify the equivalence between 72-5ptase and INPP5E, as their interpretation of loss of function depends entirely on this connection.

© 2026. Published by The Company of Biologists under the terms of the Creative Commons Attribution License (https://creativecommons.org/licenses/by/4.0/).

We thank the reviewer for raising this important point and are happy to clarify this issue.

The study by Christina Mitchell's group (2006) was among the first to characterize the protein now known as INPP5E. At that time, the protein was referred to as "72-5-phosphatase" (72-5ptase), prior to the adoption of the standardized gene/protein nomenclature INPP5E (inositol polyphosphate-5- phosphatase E). It is now well established that 72-5ptase and INPP5E refer to the same enzyme encoded by the INPP5E gene.

In that original work, the D477N substitution, then referred to the catalytic inactive 72-D480N5ptase mutant due to differences in sequence annotation and isoform numbering at the time, located within the conserved catalytic domain and was shown to abolish 5-phosphatase activity. Since then, the D477N mutation has been widely used as a catalytically inactive version of INPP5E in multiple independent studies. Importantly, subsequent publications have directly confirmed that the D477N mutation abrogates INPP5E enzymatic activity, including Cilleros-Rodriguez et al. ((2022) DOI: 10.7554/eLife.78383), which biochemically validated the loss of catalytic function.

Our INPP5E^{KD} iPSC line carries the homozygous D477N mutation and was previously characterized (ref. 18) in the context of altered SHH signaling. Given the established biochemical evidence that D477N abolishes INPP5E phosphatase activity, we interpret this allele as a functional catalytic loss- of-function model rather than a structural null.

To indicate this specific point mutation more clearly, we have included its nomenclature (D477N) also at its first appearance in the text (line 66) and in the methods section (line 307).

Additionally, prior studies from Wolfgang Baehr's group have shown that INPP5E localizes to the Golgi apparatus in the retina. The manuscript does not discuss or evaluate whether the reported phenotype could be linked to potential Golgi dysfunction, which should be addressed.

We thank the reviewer for raising the potential involvement of Golgi dysfunction.

In response, we performed additional immunofluorescence analysis in mature (d230) retinal organoids using the cis-Golgi marker GM130. Golgi morphology was quantified using ellipticity (length/width ratio), following the analytical framework described by Gupta et al. While Gupta et al. assessed overall Golgi morphology using Giantin, our analysis specifically evaluates cis-Golgi structure.

As shown in the figure included below, we did not detect statistically significant differences in GM130-labeled Golgi morphology between control and INPP5E^{KD} organoids, nor did representative images reveal overt cis-Golgi fragmentation or compaction. While this does not exclude subtle alterations in other Golgi compartments, we find no evidence for major structural disruption of the cis-Golgi.

Given that the principal phenotypes observed in our study (altered ciliary protein localization, outer segment elongation, and cytoskeletal changes) are consistent with the established role of INPP5E in ciliary phosphoinositide regulation, we consider extensive investigation of broader Golgi architecture beyond the scope of the current manuscript. Therefore, we only include this analysis below to address the reviewer's concern.

In case the editor judges it still relevant to include these data in the supplements, we can adapt.

© 2026. Published by The Company of Biologists under the terms of the Creative Commons Attribution License (https://creativecommons.org/licenses/by/4.0/).

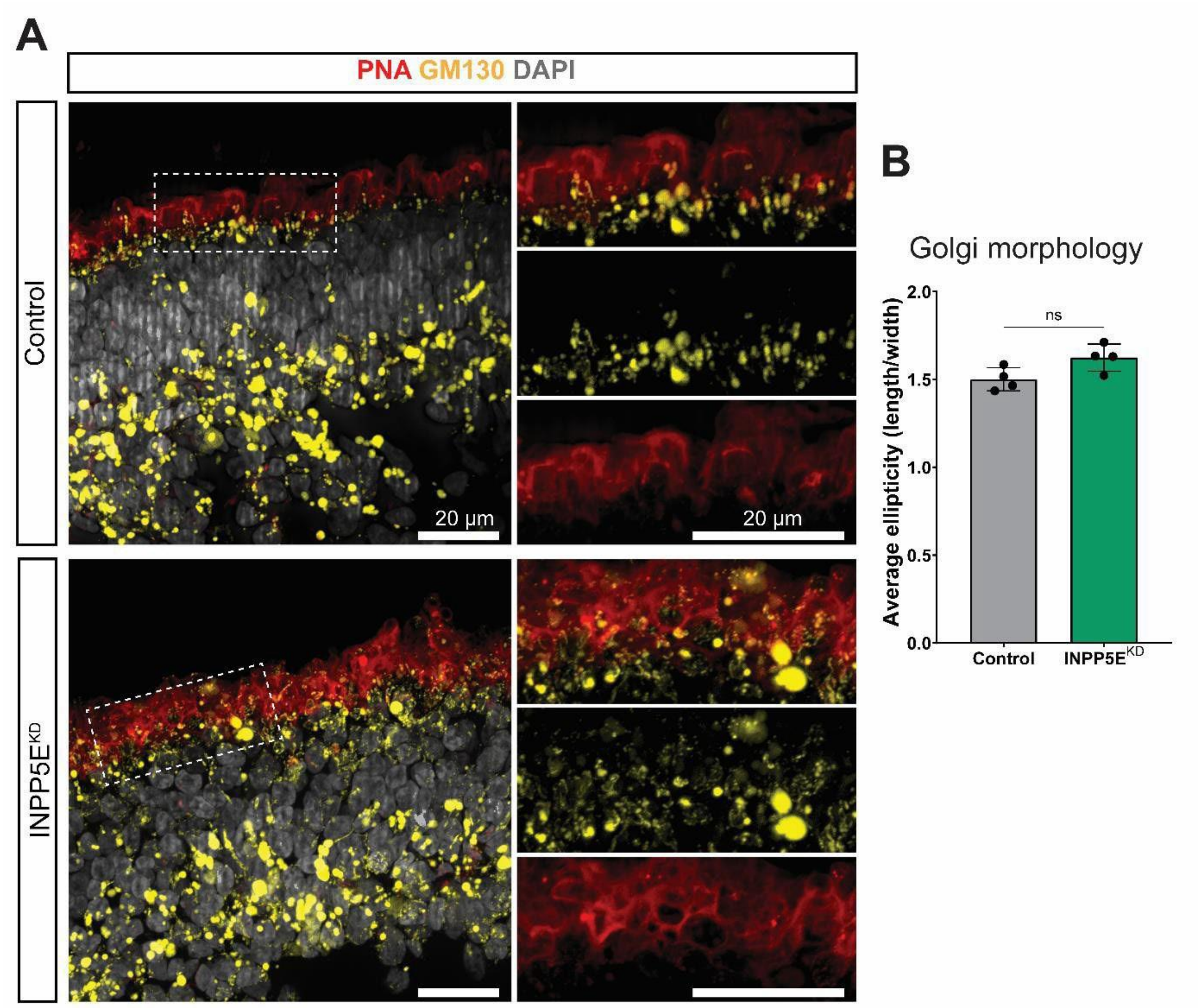

3. INPP5E deficiency is commonly evidenced by mislocalization of PIP-binding proteins such as TULP1 (also associated with RP in humans) or TULP3. Did the authors examine or observe any changes in the localization of these proteins?

We thank the reviewer for raising this important question. To address this, we have assessed potential misregulation of PIP-binding proteins by focusing on TULP3, as it is a well-established $PI(4,5)P_2$-binding protein whose ciliary localization is directly regulated by INPP5E-dependent phosphoinositide balance. We examined TULP3 localization by immunofluorescence in early-stage (d35) retinal organoids. In $INPP5E^{KD}$ organoids, we observed a significant increase in ciliary TULP3 signal compared to control organoids (Fig. S4A). This finding is consistent with previously reported accumulation of TULP3 in INPP5E-deficient systems and supports altered phosphoinositide- dependent ciliary trafficking in our model.

These data have been incorporated into the revised manuscript and included as supplementary information (Fig. S4A,C, lines 270-276).

4. Figure 1 The primary data (images) used to derive ONL length and cell counts are missing. The methods for ONL measurement and quantification are unclear: how was the ONL delineated, labeled, and counted? What controls were used in these analyses?

The ONL of retinal organoids is delineated by both a layer of densely packed nuclei, but can also be identified using phalloidin to identify the outer limiting membrane and inner limiting membrane. The ONL was then measured manually in FIJI at two separate areas per image. This is explained in the diagram below (Lines 374-375; Fig. S5).

© 2026. Published by The Company of Biologists under the terms of the Creative Commons Attribution License (https://creativecommons.org/licenses/by/4.0/).

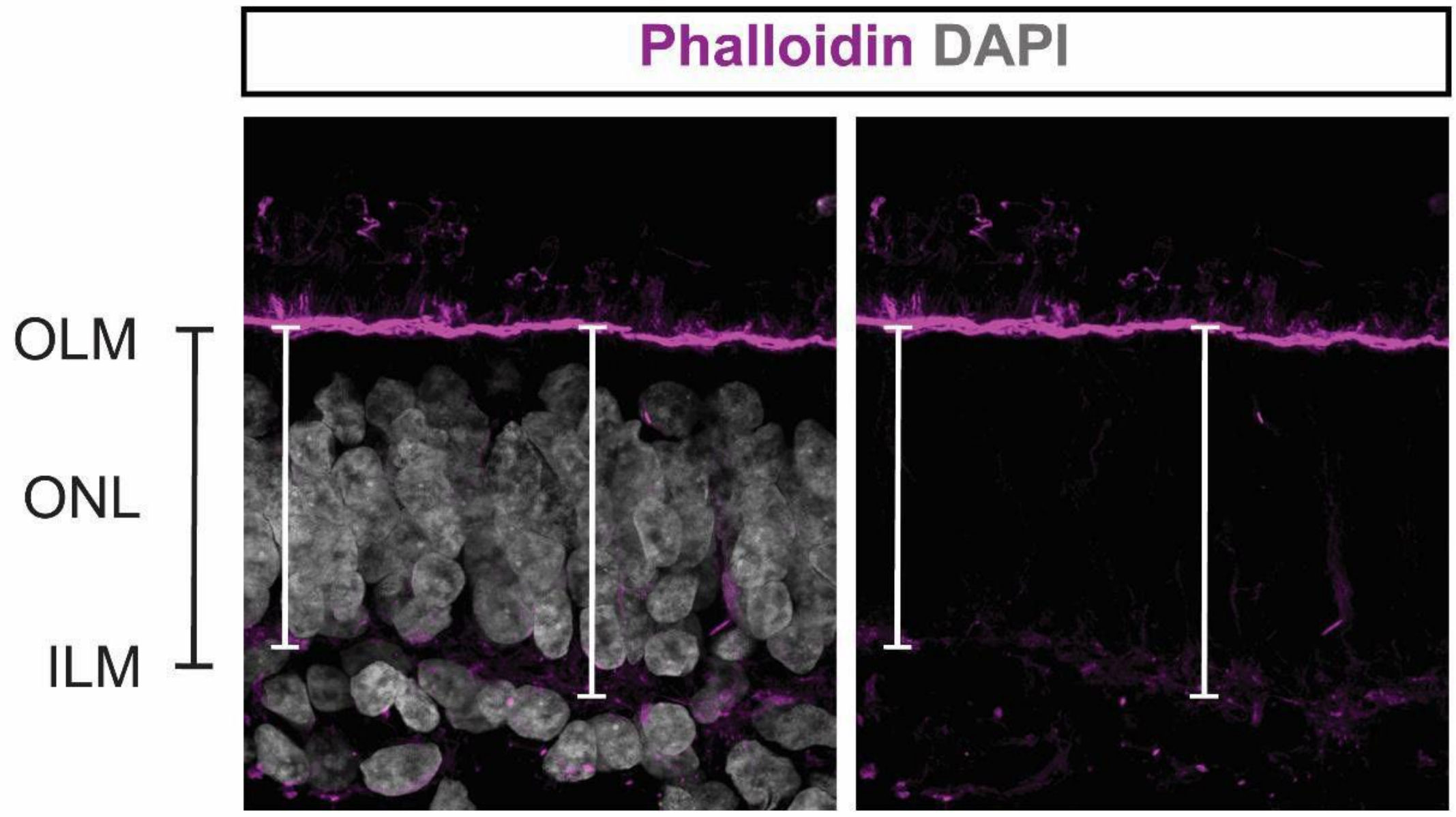

Were nuclei in other retinal layers, such as the INL, also quantified to assess specificity?

In the present study, quantification was restricted to the ONL, as our primary focus was on the role of INPP5E in photoreceptor development, connecting cilia, and outer segment membrane biogenesis. Accordingly, ONL morphology and nuclear density provide the most appropriate and directly interpretable readouts.

While inner retinal cell types are present in retinal organoids, the INL does not consistently develop the well-defined laminar organization observed *in vivo*. This structural variability limits the reliability and reproducibility of quantitative INL measurements in the organoid model.

Given the photoreceptor-specific focus of our study and the absence of evidence suggesting broad inner retinal disorganization, we prioritized detailed analysis of the ONL.

Additionally, were differences observed between rod and cone nuclei?

Direct discrimination between rod and cone nuclei was not feasible within our experimental setup. Rod and cone nuclei are not reliably distinguishable based on nuclear morphology alone in retinal organoid sections, and nuclear subtype-specific markers were not co-labeled in the DAPI-based ONL quantifications.

However, rod and cone populations were assessed at the level of mature photoreceptor differentiation using subtype-specific markers (RHO for rods and Opsin R/G for cones), as shown in Figure 5. These analyses revealed differences in total rod and cone photoreceptor numbers and outer segment morphology in $INPP5E^{KD}$ organoids. Thus, although nuclear subtype quantification was not performed, rod- and cone-specific phenotypes were evaluated using appropriate molecular markers.

Given that our primary focus was on photoreceptor outer segment structure and ciliary-associated phenotypes, subtype analysis at the level of mature photoreceptor markers provides a more biologically meaningful readout than nuclear morphology alone.

Finally, if validated, how do the authors explain the apparent increase in ONL counts observed in the absence of INPP5E?

We propose that the increased number of DAPI-positive nuclei in the ONL of $INPP5E^{KD}$ organoids reflects an early developmental alteration in progenitor dynamics rather than a late structural effect. As discussed in the manuscript (Discussion, lines 277-284), SHH signaling plays a well-established role in regulating proliferation and cell fate decisions during retinogenesis, with increased SHH activity promoting expansion of proliferative retinal progenitors.

© 2026. Published by The Company of Biologists under the terms of the Creative Commons Attribution License (https://creativecommons.org/licenses/by/4.0/).

To investigate this possibility, we examined two key SHH pathway regulators, SMO and TULP3, in early-stage (d35) retinal organoids. Both proteins displayed increased ciliary enrichment in INPP5E^{KD} organoids compared to controls (Fig. S4; lines 270-276). Given that INPP5E regulates ciliary phosphoinositide composition, which in turn modulates SHH pathway activity, these findings support a mechanistic model in which loss of INPP5E alters early SHH signaling dynamics. Such early pathway dysregulation could expand the progenitor pool and subsequently lead to the increased photoreceptor numbers observed at later stages.

While we acknowledge that more robust functional measurement of SHH pathway output would be required to conclusively establish causality, the combined developmental phenotype and ciliary localization data provide a plausible explanation for the increased ONL cell counts.

5. Figure 2 (labeled as Fig 3) The figure is titled "Delayed expression of photoreceptor precursor markers." Data are shown for day 35; however, at day 230, are recoverin and CRX staining patterns comparable between normal and mutant retinal organoids?

We thank the reviewer for suggesting this clarification.

At the mature stage (d230), we did not observe significant differences in CRX or Recoverin (RCVN) expression between control and INPP5E^{KD} retinal organoids. While early-stage (d35) organoids show a delay in photoreceptor precursor marker expression, this difference is no longer present at d230, indicating that the early developmental delay is resolved during maturation.

To address the reviewer's question directly, we have now included the d230 CRX and RCVN data as a supplementary figure (Fig. S1) and have added a corresponding description in the Results section (lines 103-104). These data support our conclusion that INPP5E loss affects early photoreceptor precursor dynamics without preventing eventual photoreceptor differentiation.

CRX-positive cells were not significantly altered at day 35 (panel B). The calculation of CRX-positive cell percentages (Line 99) is unclear—does this represent the proportion of photoreceptor precursors that are CRX positive? How were photoreceptor precursors identified or estimated?

At d35, the proportion of CRX-positive cells was not significantly different between control and INPP5E^{KD} organoids, although a slight decrease was measured (as stated in the revised manuscript, lines 100-101).

The reported values represent the percentage of total nuclei (DAPI-positive cells) that were positive for CRX. CRX is a well-established, photoreceptor lineage-defining transcription factor and is widely used as a robust nuclear marker of photoreceptor precursor cells and early photoreceptor differentiation (Kallman, A. (2020) DOI: 10.1038/s42003-020-0808-5). Accordingly, photoreceptor precursors were identified based on nuclear CRX immunoreactivity, and the percentage was calculated as the number of CRX-positive nuclei relative to the total number of nuclei within the quantified ONL region.

We have revised the text to explicitly clarify this definition and calculation (Lines 376-378).

Additionally, standard deviations (SD) are not shown.

Standard deviations (s.d.) have now been added to all relevant graphs to ensure transparent representation of data variability. The figure legends have been updated accordingly to indicate that values are presented as mean ± s.d.

6. Figure 3 (labeled as Fig 2). Did the authors observe any significant changes in INPP5E expression in the proteomics study?

As indicated with Reviewer 1, we have corrected the figure numbering to resolve the labeling error.

To answer the question: INPP5E was not detected in our bulk proteomics dataset. This is not unexpected, as data-independent acquisition (DIA)-based bulk proteomics does not consistently capture low-abundance or

© 2026. Published by The Company of Biologists under the terms of the Creative Commons Attribution License (https://creativecommons.org/licenses/by/4.0/).

poorly ionizing proteins, particularly those with restricted subcellular localization such as ciliary proteins. Therefore, absence from the dataset does not imply unchanged expression but rather reflects the sensitivity limitations of unbiased bulk proteomic analysis.

To assess INPP5E expression directly, we performed immunofluorescence analysis, which confirmed markedly reduced INPP5E signal in INPP5E^{KD} organoids at both d35 and d230 (Fig. 4; Table 1). Thus, while INPP5E abundance could not be evaluated at the proteomic level, its depletion was independently validated using targeted imaging-based analysis.

Additionally, the GO terms cell projection organization and extracellular matrix are mentioned, but their relevance to INPP5E function is not discussed. How do these pathways relate to the specific mutation studied?

The GO terms “cell projection organization” and “extracellular matrix organization” emerged from the unbiased enrichment analysis of differentially abundant proteins in mature organoids. As expected in bulk proteomic datasets, some enriched categories represent broad biological processes that encompass diverse structural and signaling components. While these categories may reflect secondary or downstream consequences of altered ciliary and cytoskeletal regulation, they are not specific to INPP5E’s established molecular function as a regulator of ciliary phosphoinositide composition.

Importantly, “cell projection organization” is a broad term that includes proteins involved in cytoskeletal remodeling and membrane extension, processes that are directly relevant to photoreceptor outer segment morphogenesis. Similarly, changes annotated under “extracellular matrix organization” may reflect alterations in tissue architecture accompanying modified photoreceptor differentiation and outer segment structure. However, these GO categories do not point to a discrete, mutation-specific signaling pathway comparable to the ciliary and SHH-related mechanisms discussed in the manuscript.

To maintain focus on the mechanistically relevant and photoreceptor-specific findings, namely altered ciliary protein localization, cytoskeletal dynamics, and outer segment membrane biogenesis, we did not expand extensively on these broader GO categories.

7. Figure 4, Structural changes in cilia are assessed using ARL13B labeling, a marker known to interact with INPP5E. To validate these findings, the authors should include additional photoreceptor ciliary markers such as acetylated tubulin, RPGR, or RP1, as PCNT does not reliably indicate ciliary morphology (lines 132-133).

We thank the reviewer for this important suggestion.

In response, we tested additional photoreceptor ciliary markers, including acetylated α-tubulin and RPGR, to further validate the observed structural changes. However, in our retinal organoid system, these antibodies did not yield reliable or reproducible ciliary staining, precluding quantitative analysis (representative images are provided below for transparency). This limitation likely reflects technical constraints inherent to organoid tissue processing and antigen accessibility rather than absence of the respective proteins.

Importantly, our ciliary analyses do not rely solely on ARL13B. We combined multiple complementary markers: ARL13B to label the ciliary membrane, GT335 (polyglutamylated tubulin) to assess axonemal microtubules, and PCNT to define the basal body. Together, these markers allow robust evaluation of ciliary number, length, and structural organization. The consistency of the phenotypes observed across membrane and axonemal markers strengthens the validity of our conclusions.

We agree that PCNT does not report on ciliary morphology per se; in our analysis it was specifically used to identify the basal body and define the proximal boundary of the connecting cilium. Morphological measurements were based on ARL13B and GT335 labeling.

While inclusion of additional photoreceptor-specific ciliary markers would further strengthen our observations, we argue that our combined use of membrane, axonemal, and basal body markers provides a robust and internally consistent assessment of ciliary structure in this system.

© 2026. Published by The Company of Biologists under the terms of the Creative Commons Attribution License (https://creativecommons.org/licenses/by/4.0/).

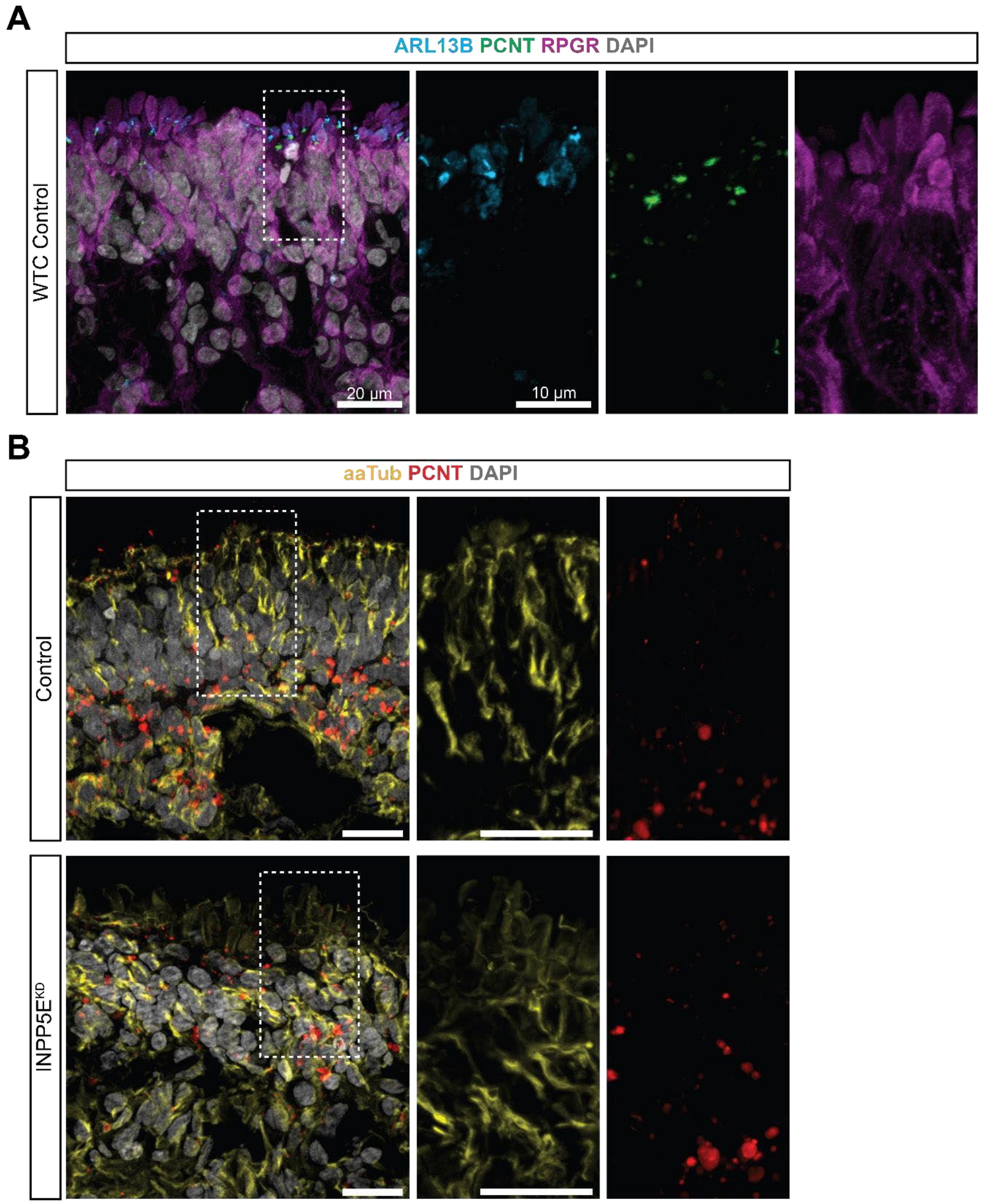

A table summarizing cilia number and length measurements at d35 and d230 would help readers better visualize the observed changes.

We thank the reviewer for this suggested addition. A table summarizing the results from the analysis has been generated as recommended (Table 1).

8. Lines 128-129: The reported values (2627 ± 1669) are unclear, do these represent colocalization coefficients (ARL13B with INPP5E)?

The reported values (e.g., 2627 ± 1669) represent the normalized mean fluorescence intensity (MFI) of INPP5E signal measured within ARL13B-positive ciliary regions. Specifically, ARL13B staining was used to define a ciliary

© 2026. Published by The Company of Biologists under the terms of the Creative Commons Attribution License (https://creativecommons.org/licenses/by/4.0/).

mask, and INPP5E fluorescence intensity was quantified within this defined area. Thus, these values do not represent colocalization coefficients, but rather the average INPP5E signal intensity restricted to the ciliary compartment.

We have revised the manuscript (lines 130-132 and 383-385) to explicitly clarify the quantification method and avoid ambiguity regarding the nature of the measurement.

Line 131: A semicolon is missing after "d230."

We apologize for this omission, the missing semicolon after "d230" has been corrected.

The variability in the data (358 ± 496) is unusually high, are the authors certain that INPP5E is being correctly detected in the mutant retinal organoids.

We are confident about our measurements. In INPP5E^{KD} organoids, INPP5E signal is markedly reduced compared to controls, consistent with loss of catalytic function. However, the D477N mutation represents a catalytic loss-of-function rather than a complete protein-null allele. As a result, low residual levels of INPP5E protein can still be detected by immunofluorescence, although at substantially reduced intensity.

Because the overall signal in INPP5E^{KD} organoids is close to background levels, small fluctuations in residual staining can result in relatively large proportional variability, which likely explains the increased standard deviation (358 ± 496). Importantly, despite this variability, the mean INPP5E signal remains significantly reduced compared to control organoids, supporting effective depletion at the protein level.

9. Figure 5 titled "Photoreceptor morphology is altered in INPP5E…", this figure presents a key conclusion of the study but lacks sufficient supporting evidence. Rhodopsin appears similarly mislocalized in both control and mutant retinal organoids, yet the authors attribute this mislocalization specifically to the mutation. Could the apparent increase in opsin-positive cells instead reflect the changes in ONL thickness noted in Figure 1?

We thank the reviewer for this careful reading and the opportunity to clarify.

First, Figure 5 addresses photoreceptor number and outer segment morphology, not rhodopsin mislocalization. The mislocalization phenotype, specifically increased rhodopsin retention in the ONL, is presented separately in Figure 7. In control organoids, a low baseline level of rhodopsin retention in the ONL is observed, which is a known feature of retinal organoid systems. However, INPP5E^{KD} organoids show a statistically significant increase in rhodopsin signal within the ONL compared to controls (Fig. 7), supporting mutation-associated trafficking alterations.

Regarding Figure 5, the primary conclusions are:

1. **an increase in the number of rod (RHO+) and cone (Opsin R/G+) photoreceptors per mm, and**
2. **a significant elongation of photoreceptor outer segments in INPP5E^{KD} organoids.**

Importantly, Figure 1 does not demonstrate an increase in ONL thickness, but rather an increase in DAPI-positive nuclei density within the ONL. As suggested in the Discussion (lines 279-283), the increased number of opsin- and rhodopsin-positive photoreceptors may indeed reflect the expanded ONL cell population observed in INPP5E^{KD} organoids. This interpretation is consistent with our proposed model of altered early progenitor dynamics.

Thus, the increase in photoreceptor number is not interpreted as an independent differentiation bias, but rather as part of the broader developmental phenotype associated with INPP5E loss.

© 2026. Published by The Company of Biologists under the terms of the Creative Commons Attribution License (https://creativecommons.org/licenses/by/4.0/).

Furthermore, the claim regarding altered outer segment (OS) length requires validation by transmission electron microscopy to substantiate the morphological findings.

We agree that transmission electron microscopy (TEM) could provide additional ultrastructural detail regarding outer segment morphology. However, we believe that TEM is not strictly required to support the conclusions presented here. Outer segment length was quantified using well-established photoreceptor markers (RHO and Opsin R/G) and measured consistently across multiple organoids and independent differentiations. The elongation phenotype was statistically significant and reproducible, supporting a robust morphological difference at the light-microscopy level.

10. Figure 6, The interpretation of changes in GT335 width is unclear. What does this alteration signify regarding photoreceptor function or morphology? The authors should clarify why INPP5E deficiency would be expected to influence this parameter and how it relates to ciliary or outer segment structure.

We are happy to provide the requested clarification.

GT335 recognizes polyglutamylated tubulin, which labels stabilized axonemal microtubules within the photoreceptor connecting cilium and the proximal region of the outer segment. In mature photoreceptors, this staining forms a characteristic ring-like structure corresponding to the axonemal core and associated microtubule architecture.

In $INPP5E^{KD}$ organoids, we observed a significant reduction in the width of GT335-positive structures, indicating altered organization of axonemal microtubule bundles (lines 184-187). This finding suggests changes in the structural integrity or spatial arrangement of the photoreceptor axoneme rather than a simple change in tubulin abundance.

Mechanistically, INPP5E regulates ciliary phosphoinositide composition, which influences cytoskeletal organization and membrane dynamics at the base of the outer segment. Disruption of this balance could plausibly affect axonemal stability and microtubule organization, consistent with the altered GT335 morphology observed (discussion, lines 243-245). Importantly, this phenotype aligns with our additional findings of altered actin organization and outer segment elongation, supporting a broader model of disrupted cytoskeletal regulation in $INPP5E^{KD}$ photoreceptors.

11. Figure 7: No clear evidence of altered opsin trafficking is apparent. Even if slight differences exist in the mutant line, these are likely indirect effects. Have the authors examined other outer segment markers beyond opsin to strengthen this conclusion?

Figure 7 specifically evaluates rhodopsin trafficking rather than general opsin localization. In Figure 7B, we quantified rhodopsin fluorescence intensity within the outer nuclear layer (ONL) and observed a statistically significant increase in $INPP5E^{KD}$ organoids compared to controls. Increased rhodopsin signal in the ONL is indicative of retention outside the outer segment and is a well- established readout of impaired rod photoreceptor trafficking.

Importantly, this phenotype is subtype-specific. As shown in Supplementary Figure S3, cone photoreceptors labeled with Opsin R/G do not exhibit a comparable increase in ONL-associated signal. The absence of altered cone opsin retention argues against a global or indirect trafficking defect and instead supports a rod-specific impairment in rhodopsin transport.

While additional outer segment markers could further characterize trafficking dynamics, we argue that the statistically significant increase in ONL rhodopsin retention, together with preserved cone opsin localization, provides strong evidence for a specific trafficking alteration associated with INPP5E deficiency rather than a nonspecific secondary effect.

Importantly, ARL13B does not colocalize with opsin, indicating that ARL13B is not localized to the outer segment. The apparent increase in ARL13B-positive vesiculation suggests possible membrane trafficking abnormalities, which should be further examined by higher-resolution TEM imaging.

© 2026. Published by The Company of Biologists under the terms of the Creative Commons Attribution License (https://creativecommons.org/licenses/by/4.0/).

In Figure 7, ARL13B was co-stained with peanut agglutinin (PNA), a well-established marker of the photoreceptor outer segment membrane. Quantitative analysis demonstrates a significant increase in ARL13B signal within the PNA-positive outer segment region in INPP5E^{KD} organoids compared to controls. This indicates a redistribution of ARL13B from its typical localization at the connecting cilium toward the outer segment compartment in the absence of functional INPP5E.

We agree that altered ARL13B localization may reflect changes in membrane trafficking dynamics, and we discuss this possibility in the manuscript (Discussion, lines 254-259). Importantly, the observed redistribution is supported by quantitative colocalization within a defined outer segment mask rather than qualitative impression alone.

While higher-resolution ultrastructural analysis by TEM could provide additional insight into membrane organization within the outer segment, the current immunofluorescence-based data are sufficient to demonstrate altered ARL13B compartmentalization.

12. Supplementary Fig 1, rather than NPHP1, a basal body marker, the authors should include additional ciliary markers as recommended earlier to provide a more comprehensive assessment of ciliary structure.

As noted in our earlier response, we attempted to include additional ciliary markers, including acetylated α-tubulin, to further characterize ciliary structure. However, in our retinal organoid system, these antibodies did not yield reliable or reproducible ciliary staining, precluding meaningful quantitative analysis. Representative images are provided above for transparency.

Due to limited availability of mature organoid tissue during the rebuttal phase after providing significant additional experimental data requested by the reviewer, further antibody testing and optimization were not feasible. Importantly however, our assessment of ciliary structure does not rely solely on NPHP1. We used complementary markers including ARL13B (ciliary membrane), GT335 (polyglutamylated axonemal tubulin), and PCNT (basal body), which together allow robust evaluation of ciliary number, length, and structural organization.

While inclusion of additional markers would further expand the characterization, we argue that our informative set of markers provides a consistent and internally validated assessment of ciliary architecture in INPP5E^{KD} organoids.

Minor comments

Figures 2 and 3 are mislabeled. Fig. 3 should be Fig. 2 and vice versa.

Figure order of figures 2 and 3 have been corrected in the revised draft.

Authors repeatedly use the term "outer segment stability." What is meant by stability of OS? This term needs to be defined. Sometimes this is referred to as "OS disc stability."

We thank the reviewer for highlighting this point.

In the original manuscript, the term “outer segment stability” was used to describe findings from previously published INPP5E mouse models in which loss of INPP5E resulted in shortened outer segments and disrupted disc morphology, reflecting compromised structural integrity of outer segment discs.

To improve clarity and precision, we have replaced this terminology with the more specific term “outer segment disc stability” in the revised manuscript (lines 60, 220, and 229). This wording more accurately reflects the structural integrity of stacked outer segment discs rather than general outer segment morphology.

Second decision letter

© 2026. Published by The Company of Biologists under the terms of the Creative Commons Attribution License (https://creativecommons.org/licenses/by/4.0/).

MS ID#: jcs.264431R1

MS TITLE: Loss of INPP5E affects photoreceptor outer segment membrane biogenesis in iPSC-derived human retinal organoids

AUTHORS: Kae R Whiting; Mariam G Aslanyan; Lynn van Summeren; Tina Beyer; Katrin Dahlke; Karsten Boldt; Ronald Roepman
ARTICLE TYPE: Research Article

Dear Kae and Ronald,

We have now reached a decision on the above manuscript. Happy almost acceptance ;)

As you will see, the reviewers gave favourable reports but one critical point requires text edits to your manuscript in order for me to accept it. I hope that you will be able to carry these out quickly so we can accept your paper, without further comments from reviewers.

Add on line 57 a mention of LCA, as requested with a reference (Sangermano et al (2021). Broadening INPP5E phenotypic spectrum: detection of rare variants in syndromic and non-syndromic IRD. npj Genomic Medicine. 6. 53. 10.1038/s41525-021-00214-8.; Wang et al. (2013) Comprehensive molecular diagnosis of 179 Leber congenital amaurosis and juvenile retinitis pigmentosa patients by targeted next generation sequencing. J Med Genet. 2013 Oct;50(10):674-88. doi: 10.1136/jmedgenet-2013-101558; Wang et al 2015 Comprehensive Molecular Diagnosis of a Large Chinese Leber Congenital Amaurosis Cohort. Investigative Ophthalmology & Visual Science June 2015, Vol.56, 3642-3655. doi:https://doi.org/10.1167/iovs.14-15972) For example…

Tone down the antimouse statement lines 61-64 and line 206-207- this is what the reviewer 2 dug into in the revision. I think the issue is that the retinal specific deletion in Gupta et al. (2025) does look like LCA (See above) causing rapid PR loss early. (Inpp5e is crucial for photoreceptor outer segment maintenance. J Cell Sci. 2025 Feb 15;138(4):JCS263814. doi: 10.1242/jcs.263814). Perhaps clarify a little on the role in slower degeneration like human RP, thus need for a different (human) model, if that is the distinction you are making? However, in the Discussion, line 294 you say it models only early retinal development, so that might not work which is why they say the mouse performs better. Given all this, I would suggest you instead delete lines 61-64 and edit the discussion lines 205-208, emphasizing this is the first time this is done in human retinal organoids and how the role for INPP5E in human neuroretina development is unknown in a growing spectrum of IRDs.

Reviewer 1

The authors addressed all my comments. It can be accepted for publication.

Reviewer 2

The authors have made extensive effort to address the concerns raised in the previous round and I appreciate it.

My impressions on the revised work are below:

Minor: Is there a reason not to mention in the Introduction that INPP5E mutations are associated with severe, early-onset degenerative disease, such as LCA? The mouse model appears to recapitulate the severe degeneration observed in LCA patients. While I agree that mouse models do not always reflect the human phenotype, in the case of INPP5E, they seem to perform better than organoids. The authors have addressed this to some extent in the Discussion.

© 2026. Published by The Company of Biologists under the terms of the Creative Commons Attribution License (https://creativecommons.org/licenses/by/4.0/).

The increased number of nuclei could reflect reduced cell death in KD ROs. This possibility should be acknowledged in the Discussion.

CRX-positive nuclei were reduced, but not significantly. This limitation is not discussed. Moreover, the data presented do not support the conclusion that INPP5E plays a role in early differentiation.

Changes in rod or cone outer segment morphology based on single-marker analysis and immunofluorescence are not convincing. The authors should be more cautious in their interpretation and clearly state the limitations of the study.

Second revision

Author response to reviewers' comments

Point-by-point response to the Reviewers' comments

Please note that the indicated line numbers refer to the clean text manuscript file.

We thank the Editor and Reviewer 2 for their constructive feedback and valuable suggestions, which have helped us further improve the manuscript. All points raised have been carefully addressed below. We also thank Reviewer 1 for their recommendation to accept the revised manuscript.

From the Editor:

- Add on line 57 a mention of LCA, as requested with a reference (Sangermano et al (2021). Broadening INPP5E phenotypic spectrum: detection of rare variants in syndromic and non-syndromic IRD. npj Genomic Medicine. 6. 53. 10.1038/s41525-021-00214-8.; Wang et al. (2013) Comprehensive molecular diagnosis of 179 Leber congenital amaurosis and juvenile retinitis pigmentosa patients by targeted next generation sequencing. J Med Genet. 2013 Oct;50(10):674-88. doi: 10.1136/jmedgenet-2013-101558; Wang et al 2015 Comprehensive Molecular Diagnosis of a Large Chinese Leber Congenital Amaurosis Cohort. Investigative Ophthalmology & Visual Science June 2015, Vol.56, 3642-3655. doi:https://doi.org/10.1167/iovs.14-15972) For example…

Response:
We appreciate the recommendation and have included Leber congenital amaurosis as a phenotype present in individuals with variants in INPP5E, including the suggested references (lines 60-62).

- Tone down the antimouse statement lines 61-64 and line 206-207- this is what the reviewer 2 dug into in the revision. I think the issue is that the retinal specific deletion in Gupta et al. (2025) does look like LCA (See above) causing rapid PR loss early. (Inpp5e is crucial for photoreceptor outer segment maintenance. J Cell Sci. 2025 Feb 15;138(4):JCS263814. doi: 10.1242/jcs.263814). Perhaps clarify a little on the role in slower degeneration like human RP, thus need for a different (human) model, if that is the distinction you are making?

However, in the Discussion, line 294 you say it models only early retinal development, so that might not work which is why they say the mouse performs better. Given all this, I would suggest you instead delete lines 61-64 and edit the discussion lines 205-208, emphasizing this is the first time this is done in human retinal organoids and how the role for INPP5E in human neuroretina development is unknown in a growing spectrum of IRDs.

Response:
Thank you for the feedback regarding adjusting the phrasing of the mouse and organoid comparisons in the manuscript. As recommended, we have revised the text to soften the critical statements about the mouse models and to more clearly describe their phenotypes, while still explaining why it is of particular interest that we, for the first time, studied INPP5E loss in a human-based model system (the retinal organoids) (lines **63-72** and **214-219**). We believe these changes now better balance the novelty and

© 2026. Published by The Company of Biologists under the terms of the Creative Commons Attribution License (https://creativecommons.org/licenses/by/4.0/).

importance of using retinal organoids in our study with appropriate acknowledgment of the key findings from previously published mouse models.

From Reviewer 2:

- Minor: Is there a reason not to mention in the Introduction that INPP5E mutations are associated with severe, early-onset degenerative disease, such as LCA? The mouse model appears to recapitulate the severe degeneration observed in LCA patients. While I agree that mouse models do not always reflect the human phenotype, in the case of INPP5E, they seem to perform better than organoids. The authors have addressed this to some extent in the Discussion.

Response:

We thank the reviewer for the recommendation. While we initially did not include LCA in the manuscript due to the relatively low prevalence of LCA in individuals with pathogenic variants in INPP5E, we have since included it to the introduction (lines **60-62**). Additionally, we have revised the comparison of mouse and human model systems to reflect a fairer comparison, both in the introduction (lines **65-69**) and the discussion (lines **217-219**).

- The increased number of nuclei could reflect reduced cell death in KD ROs. This possibility should be acknowledged in the Discussion.

Response:

We thank the reviewer for this helpful suggestion. We agree that reduced cell death could, in principle, contribute to the increased number of nuclei observed in mature $INPP5E^{KD}$ ROs. We have now acknowledged this possibility briefly in the Discussion (Lines **301-305**) as requested. However, because we did not directly assess cell death in the present study, we feel that further speculation on this mechanism would not be warranted. Moreover, previous studies of INPP5E loss in the retina report rapid retinal degeneration, outer segment defects, and photoreceptor cell loss rather than improved cell survival. We therefore consider altered developmental signaling and/or progenitor dynamics a more likely explanation for the increased nuclei, while recognizing that altered cell survival cannot be excluded without direct analysis.

Added to the discussion (lines **301-305**):

“Although our findings support a role for INPP5E in signaling regulation and/or progenitor dynamics during early RO development, we cannot exclude that altered cell survival, including reduced cell death in $INPP55E^{KD}$ ROs, might contribute to the phenotype, as cell death was not directly assessed in this study. Future work to assess the role of SHH signaling during retinogenesis will help elucidate this hypothesis further.”

- CRX-positive nuclei were reduced, but not significantly. This limitation is not discussed. Moreover, the data presented do not support the conclusion that INPP5E plays a role in early differentiation.

Response:

We thank the reviewer for this important comment. We agree that the reduction in CRX-positive nuclei was not statistically significant and have now acknowledged this more clearly as a limitation. Our original interpretation was based on the combination of reduced CRX-positive nuclei and significantly reduced RCVN expression during early RO differentiation. However, we agree that these data do not prove a direct role for INPP5E in early differentiation. We have therefore softened the conclusion and now state that INPP5E loss is associated with altered early photoreceptor marker expression.

Added to the discussion (lines **278-283**):

“In addition, INPP5E loss seemingly led to a delay in photoreceptor precursor cells in early stage ROs,

© 2026. Published by The Company of Biologists under the terms of the Creative Commons Attribution License (https://creativecommons.org/licenses/by/4.0/).

marked by the decrease in RCVN expression and CRX expressing cells, both of which were rescued by the time the ROs reached maturation at d230. However, this early differentiation phenotype should be interpreted with caution, as the reduction in CRX-positive nuclei did not reach statistical significance. Therefore, these data suggest altered expression of early photoreceptor markers but do not establish a direct role for INPP5E in early retinal differentiation."

- Changes in rod or cone outer segment morphology based on single-marker analysis and immunofluorescence are not convincing. The authors should be more cautious in their interpretation and clearly state the limitations of the study.

Response:
We thank the reviewer for this important comment. We agree that immunofluorescence analysis does not provide the same ultrastructural resolution as electron microscopy and that this limitation should be stated more clearly. However, our conclusion that INPP5E loss affects photoreceptor outer segment organization is not based solely on single-marker analysis of rod or cone markers. Rather, it is supported by multiple complementary observations, including altered morphology of RHO- and Opsin-positive structures, increased rhodopsin localization within the ONL, altered ARL13B localization in PNA-positive outer segment regions, and changes in actin- and tubulin-associated structures. We have therefore revised the Discussion to clarify the limitations of immunofluorescence-based analysis while maintaining that the combined data support an effect of INPP5E loss on photoreceptor outer segment organization.

Added to the discussion (lines **230-235**):

"The changes observed in RHO- and Opsin- positive structures, as well as the affected ARL13B distribution and rhodopsin localization, support the conclusion that INPP5E loss affects photoreceptor outer segment organization in ROs. However, it is important to note that immunofluorescence analysis lacks the ultrastructural resolution of outer segment disc organization. Thus, further ultrastructural analysis will be valuable to further define the precise nature of these OS defects."

Third decision letter

MS ID#: jcs.264431R2

MS Title: Loss of INPP5E affects photoreceptor outer segment membrane biogenesis in iPSC-derived human retinal organoids

Authors: Kae R Whiting; Mariam G Aslanyan; Lynn van Summeren; Tina Beyer; Katrin Dahlke; Thomas Theil; Karsten Boldt; Ronald Roepman
Article Type: Research Article

Dear Kae and Ronald,

CONGRATULATIONS! I am thrilled to accept your submission for publication in Journal of Cell Science, pending standard publication integrity checks.

© 2026. Published by The Company of Biologists under the terms of the Creative Commons Attribution License (https://creativecommons.org/licenses/by/4.0/).
